# Bibliometrics beyond citations: introducing mention extraction and analysis

**DOI:** 10.1007/s11192-024-05116-x

**Published:** 2024-08-02

**Authors:** Eugenio Petrovich, Sander Verhaegh, Gregor Bös, Claudia Cristalli, Fons Dewulf, Ties van Gemert, Nina IJdens

**Affiliations:** 1 Department of Philosophy and Education Sciences, University of Turin, Turin, Italy; 2 Department of Philosophy, University of Tilburg, Tilburg, The Netherlands

**Keywords:** Mention extraction, Mention analysis, History of science, EDHIPHY, History of philosophy

## Abstract

Standard citation-based bibliometric tools have severe limitations when they are applied to periods in the history of science and the humanities before the advent of now-current citation practices. This paper presents an alternative method involving the extracting and analysis of *mentions* to map and analyze links between scholars and texts in periods that fall outside the scope of citation-based studies. Focusing on one specific discipline in one particular period and language area—Anglophone philosophy between 1890 and 1979—we describe a procedure to create a *mention index* by identifying, extracting, and disambiguating mentions in academic publications. Our mention index includes 1,095,765 mention links, extracted from 22,977 articles published in 12 journals. We successfully link 93% of these mentions to specific philosophers, with an estimated precision of 82% to 91%. Moreover, we integrate the mention index into a database named EDHIPHY, which includes data and metadata from multiple sources and enables multidimensional mention analyses. In the final part of the paper, we present four case studies conducted by domain experts, demonstrating the use and the potential of both EDHIPHY and mention analyses more generally.

## Introduction

The publication of the science citation index (SCI) by Eugene Garfield in 1964 revolutionized the way in which we study and analyze science. Prior to the advent of SCI, bibliometric studies were confined to small datasets (Bradford, 1934; Lotka, 1926) and statistics on scientific activity were limited to the scientific workforce of individual countries (Godin, 2005). Thanks to the SCI, science analysts gained unprecedented access to the *fabric* of science. Citations offered researchers a way to visualize links between people, ideas, journals, and institutions (Elkana et al., 1978; Leydesdorff & Amsterdamska, 1990; Small, 2003; Wouters, 1999). The discipline of scientometrics developed around the notion of citation and the infrastructure of SCI, which, for several decades, stood as the sole source of citation data (De Bellis, 2014; Mingers & Leydesdorff, 2015).

Sixty years after the first publication of the SCI, data on science and scientific activity has exploded. Not only have new citation databases emerged as competitors to Web of Science, the successor to SCI and its associated databases, but the industry of science analytics has swollen enormously. New databases such as Dimensions aim to encompass the entire scientific cycle, from grant proposals to publications up to policy documents, clinical trials and patents, and integrate a wide range of linkage data, from citations to online mentions in social media (Herzog et al., 2020). The application of advanced artificial intelligence systems on this data has been heralded as the next scientific revolution, accelerating new discoveries based on the automated analysis of huge datasets of scientific publications (The Economist, 2023).

This ‘big data’ revolution has an important limitation though: existing databases cover only a small segment of the history of science and the humanities. While many academic disciplines are hundreds or, in some cases, even thousands years old, these databases cover only their most recent history. According to Clarivate, Web of Science reference links for records in the natural and social sciences go back to the beginning of the twentieth century (Birkle et al., 2020) but indexing results are reliable only from 1980 onwards (Sugimoto & Larivière, 2018). Similarly, Scopus claims that its records go back to 1788, but its data only seem reliable from 1996 onwards (Sugimoto & Larivière, 2018). Dimensions’ promise to achieve full coverage of scholarly and scientific literature, finally, has not yet been fully realized either. Experiments with the database show that data for records before the 1990s remain limited or incomplete—though its coverage is rapidly improving.

Considering the limited coverage and reliability of data concerning the history of science and the humanities, any quantitative analysis that aims to cover periods before the 1980s must turn to other sources, such as archives of digitized texts like Google Books or JSTOR (Ramsey & Block, 2022). These sources, however, do not provide *relational data*, in the form of links between documents, which are the hallmark of citation databases and which enable the application of advanced science mapping techniques such as citation networks, co-citation analysis or bibliographic coupling (Börner et al., 2005; Petrovich, 2021b; Waltman & van Eck, 2014). From the viewpoint of scientometrics, then, the kinds of analyses that can be performed on these datasets are rather limited.

What is more, the possibility of extending scientometric analyses to the more distant past does not only depend on the *coverage* of citation databases. The extension of citation analysis is constitutively limited by the fact that current citation practices are a relatively recent invention in scientific communication (Small, 2010). Citations in their modern format emerged only in the beginning of the twentieth century, when referencing within scientific journals stabilized and was progressively standardized (Bazerman, 1988; Csiszar, 2018; Gross et al., 2002).^1^ Citation analysis as such, therefore, is not suited to study most of the history of science, simply because of the sheer lack of citations (Leydesdorff & Wouters, 1999).

The practice of *referencing*, intended as acknowledging the sources of external contributions incorporated into texts, whether these sources are other texts or other scholars, is much older than the invention of modern citations: it can be traced back to the earliest stages of scholarship. Aristotle (384–322 BC), for example, already referred to predecessors like Democritus and Empedocles in his *Metaphysics.* This opens up the possibility to apply a more generalized form of citation analysis to the entire history of science and scholarship, if a suitably wider concept of reference is used.

In particular, we can distinguish several textual devices that authors adopt to realize the act of referencing. These devices generate different types of references. In contemporary scientific communication, the key device is the *citation*. A citation is a link between two *documents,* which is established via adding a cited reference to the citing document (Van Raan, 2019; Wouters, 1999). The cited reference contains all relevant information to identify the cited document, realizing the “manifest intertextuality” of scholarly writing (Hyland, 1999). A different device is the *mention*. A mention is a link *between a document and a person*, which is established by the occurrence of a *proper name* in the mentioning document. Consider, for instance, the following passage:

After Leibnitz attention is more and more directed towards our knowledge of time, how it is possible and what it involves, this phase reaching its theoretical completion in Kant. While Leibnitz held that time in us is only possible if there be a real succession, Kant insists on the other hand that the knowledge of succession presupposes that of time. (McIntyre, 1895, p. 339)

In this passage, the author refers to both Gottfried Leibniz (1646–1716) and Immanuel Kant (1724–1804), but the references are made via the philosophers’ proper names, without a formal citation to a book or document. Mentions are thus a form of reference that does not point to documents but directly to persons.^2^

In this paper, we introduce a new method, called *mention extraction*, that aims to extract this different textual device for referencing from a corpus of texts. The great advantage of mentions over citations is that mentions were the most important type of reference before citations were invented. For almost the entire history of the sciences and the humanities, from Antiquity to today, mentions have represented a key mechanism to establish connections between ideas and carry forward the dialogue between scholars (Connors, 1998, 1999). In many disciplines of the humanities, mentions are still widely used along with standard citations. And even in contemporary science, the presence of eponyms, such as “Einstein’s theory of relativity” or “Alzheimer’s disease”, attests that mentions have still some currency in the age of citations (Cabanac, 2014; Merton, 1957; Thomas, 2016). Contrary to citation analysis, which is limited to the most recent period of science and the humanities, *mention analysis* can, in principle, be applied to their entire history. Moreover, it is suitable for proto-citations or not fully standardized citations, as long as they include the name of the cited author.

In this paper, we describe the method for creating a *mention index* starting from a collection of scholarly documents. The method consists of two parts: first, the identification and extraction of mentions, and second, the linkage of mentions with their proper referents. To demonstrate the effectiveness and potential of this method, we explain how we applied it to build a mention index from a corpus of publications in philosophy, a discipline that did not reliably use standardized citations up until the 1970s. The mention index that we built constitutes the core of a database called EDHIPHY, acronym for ‘Enriched Data for the HIstory of PHilosophY**’**, which integrates relational mention data with a wider set of data about philosophy. EDHIPHY allows rich, multidimensional quantitative analyses of philosophy that would otherwise be impossible or extremely limited in scope. In the second part of the paper, we demonstrate what kind of investigations the mention index of EDHIPHY makes possible, using mention analysis to address key questions about the development of twentieth-century philosophy. This application of our method to a concrete case shows that the extraction of mentions and their quantitative analyses can be a powerful method for investigating periods in the history of science and the humanities that were, until now, out of reach for standard citation analysis.

The rest of the paper is organized as follows. In the next section, we present the method of extracting mentions and building a mention index. This method consists of three steps: first, the preparation of the texts (Sect. “Preparation of texts”), second, the identification and extraction of mentions (Sect. “Identification of mentions”), and last, the process of linking mentions to their proper referents (Sects. “Creation of the entity ruler”-“Linking mentions to philosophers”). In Sect. "Evaluation of linking”, we assess the reliability of EDHIPHY’s mention index by means of a statistical evaluation of the linking process. In the following section of the paper (Sect. “EDHIPHY and mention analysis in action”), four case studies are developed using EDHIPHY’s mention data. These four case studies show how mentions can be used to measure intellectual success (Sect. “Quantity of mentions as proxy for intellectual success”), to trace trends over time (Sect. “Mention statistics tracking temporal shifts in intellectual success”), to explore the reception of particular academics in different institutions (Sect. “Comparative analysis to differentiate reception at different institutions”), and to map the changing structure of an academic community via co-mention networks (Sect. “Co-mention analysis”). Sect. “Concluding remarks” concludes with some remarks on the advantages and limitations of mention analysis in comparison with citation analysis.

## Building the mention index

Throughout the history of Western philosophy, dialogue and discussion among philosophers have been decisive dimensions of philosophical practice. It was already common for philosophers in Ancient Greece to discuss the work of fellow philosophers: several figures in Platonic dialogues are based on Plato’s contemporaries and Aristotle offered an overview of the metaphysics of his predecessors in the book Alpha of his own *Metaphysics*. Long before the invention of citations, the writings of philosophers were interwoven through a dense web of direct and indirect references to other philosophers, scientists, artists, and intellectuals (Connors, 1998, 1999).

These references occur in the form of what we call *mentions.* Roughly speaking, there are two types of mentions in philosophy. *Direct* mentions refer to philosophers by a proper name, such as a surname (e.g., “Descartes’ concept of *res cogitans*”), a full name (e.g., “Bertrand Russell argues that…”), or a nickname (e.g., “According to the Stagirite…”, referring to Aristotle), and are thus easily identifiable by the reader. *Implicit* mentions, by contrast, refer to a philosopher without explicitly mentioning them in the text. A competent reader can identify them using background knowledge or the hints in the text but not on the basis of proper names.

In principle, both direct and implicit mentions can be extracted from a corpus of text. Direct mentions, however, are the most suitable for the kind of automatic extraction that can be implemented by an algorithm and therefore can easily be scaled up. Implicit mentions, by contrast, require a substantial level of background knowledge, which is challenging to infuse into an algorithm. Our mention extraction methodology, accordingly, focuses on direct mentions only. They are operationally defined as occurrences of *proper names* referring to philosophers within a document.

Creating a mention index involves three tasks. First, a corpus of texts where mentions occur must be built. Second, mentions need to be identified and extracted from the texts. Third, the mentions must be attributed to their proper referents, appropriately solving cases of *homonymity* (e.g., a mention to “Marx” could refer to either Karl Marx or Werner Marx) and *synonymity* (e.g., the mentions “R. Carnap”, “Rudolph Carnap” and “Carnap” should all be attributed to Rudolf Carnap). The following sections provide a detailed discussion of how we executed these tasks in the building of EDHIPHY’s mention index. As we highlighted before, the same method can be applied to any other field or period where mentions play a relevant role.

### Preparation of texts

In its current version, EDHIPHY’s mention index focuses on a particular segment of the history of philosophy, namely Anglophone philosophy from the late nineteenth century to the latter half of the twentieth century. The corpus used for creating it includes 22,977 articles published in 12 philosophy journals between 1890 and 1979, for a total of around 115 million words (Table 1). The 12 journals were selected based on the central role that they played in the development of Anglophone philosophy.^3^ Their publications mirror the evolution of the discipline within the English-speaking world, spanning from the late nineteenth century through the latter half of the twentieth century.^4^

**Table 1:** Descriptive statistics of the corpus used to build EDHIPHY’ mention index

Journal	Articles	Incidence in the corpus	PY of the earliest article	Avg length (in words)	Articles without author (%)	Multi-author articles (%)
Analysis	1293	6%	1933	2150.2	1%	5%
Mind	3480	15%	1890	4888.0	8%	2%
Philosophical studies	1035	5%	1950	3168.5	1%	5%
Philosophy	1627	7%	1926	5071.8	1%	6%
Philosophy and phenomenological research	1749	8%	1940	5068.8	1%	2%
Philosophy of science	1623	7%	1934	5419.2	1%	6%
Proceedings of the aristotelian society	1060	5%	1890	8049.0	0%	5%
Synthese	1254	5%	1936	5601.9	5%	4%
The journal of philosophy	4494	20%	1904	3987.8	1%	2%
The monist	2171	9%	1890	5996.9	2%	3%
The philosophical quarterly	819	4%	1950	5361.7	2%	2%
The philosophical review	2372	10%	1892	5981.7	3%	1%
*All journals aggregated*	*22,977*	*100%*	*1890*	*4964.1*	*3%*	*3%*

Italics are used for rows of tables describing totals

EDHIPHY focuses on research articles, excluding other types of publications such as book reviews, editorials, critical notices, and other minor document categories. Nevertheless, the same methodology can be applied to any kind of document, including books and unpublished texts such as archival materials, insofar as they are in machine-readable form. The full-texts and metadata of the articles were obtained from JSTOR through its Data for Research service (Burns et al., 2009).

**Fig. 1:**
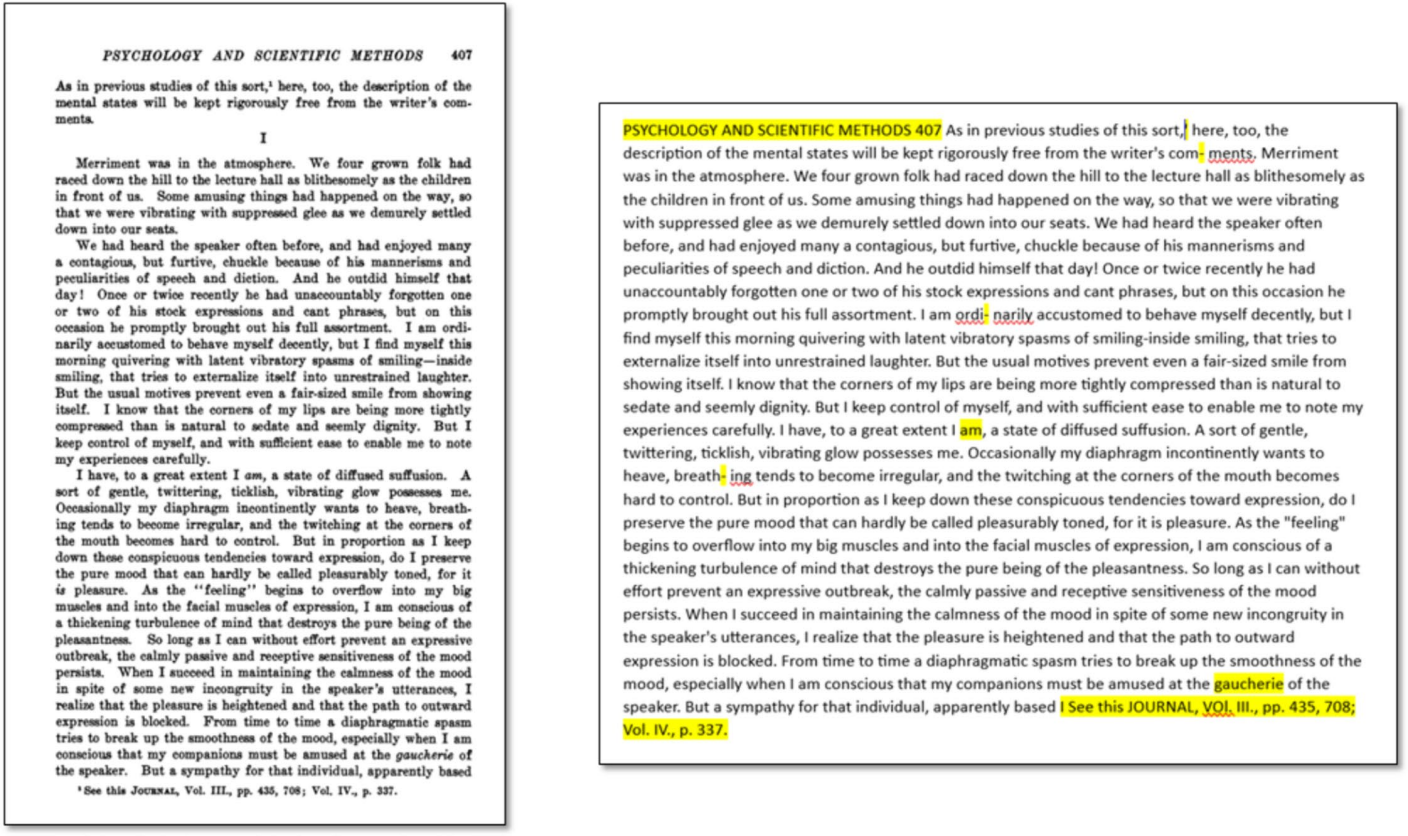
PDF of the original document (on the left) vs text captured with OCR provided by JSTOR (on the right). Mistakes and losses in text formatting and page layout are highlighted in yellow. Note the header conflated with the main text, the loss of the section marker, the superscripts wrongly captured, the words artificially split, the removal of the italics, and the footnote that is attached to the middle of a main text’s sentence, breaking the syntax of the sentence

The full-text data provided by JSTOR results from the application of Optical Character Recognition (OCR) technology to scanned pages of the documents. This process is not perfect and produces both mistakes and loss of information. In particular, errors in the OCR affect accented letters, letters with the German *Umlaut*, non-Latin characters such as Greek letters, superscripts and subscripts, and formal notation used in mathematical and logical formulas. The loss of information, on the other hand, affects both text formatting and page layout. Typographic styles such as italics or variations in font size are not accurately preserved. Headers, footnotes, section markers, section titles, page numbers, formulas, quotes, and indentations are all conflated in the same batch of text (see Fig. 1). Any distinction between the main text and para-texts such as the title, byline, and bibliography is lost. In sum, it is almost impossible to restore the original, rich structure of a document from textual data as provided by JSTOR.

**Fig. 2:**
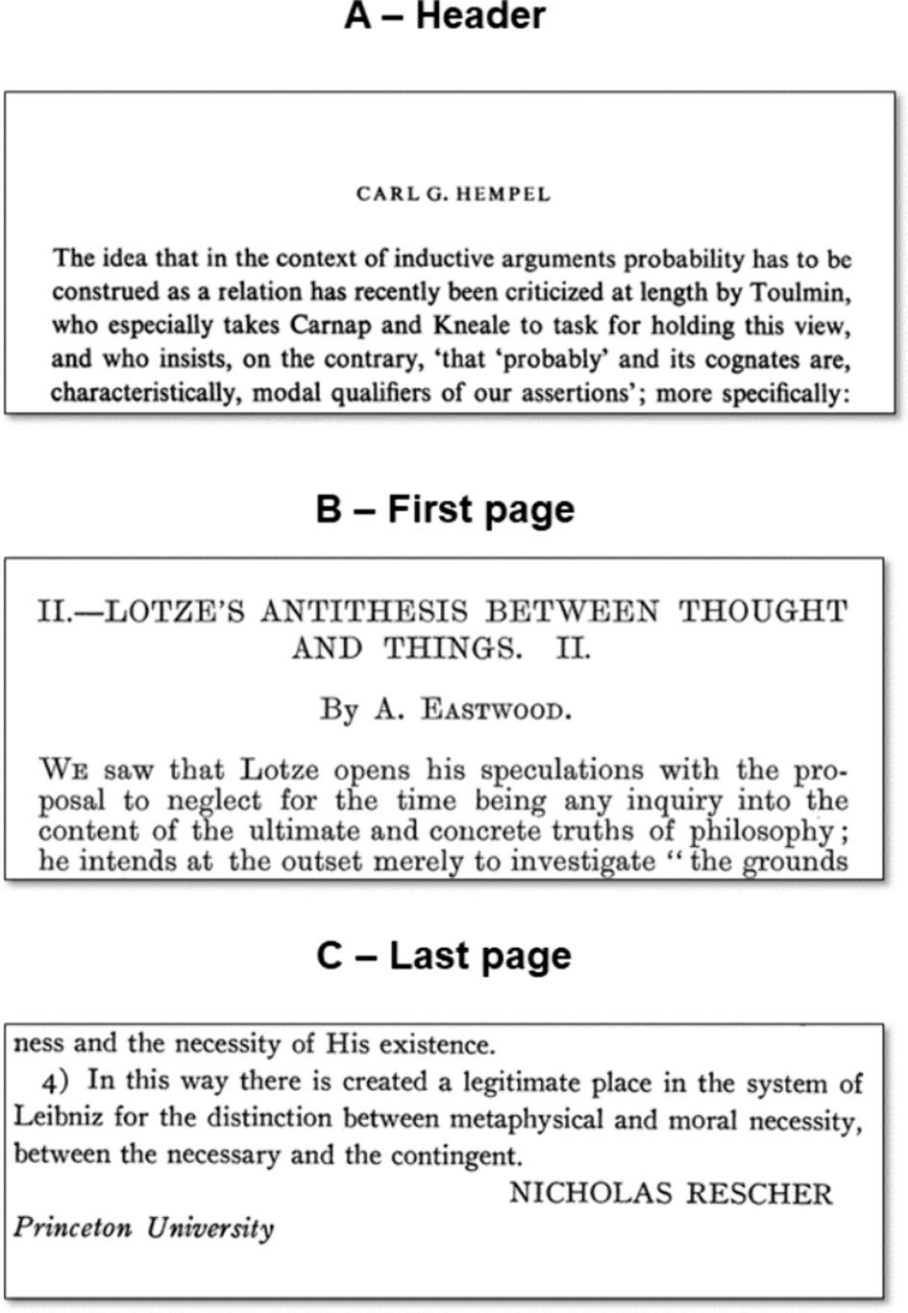
Examples of author names that generate false self-mentions if not removed

For text analysis methods relying on a bag-of-word approach such as classic topic-modelling, these issues are to a large extent irrelevant, as they do not substantially compromise the final results (Malaterre & Lareau, 2022). Mention extraction, however, demands high-quality textual data, as untreated data can easily generate artifacts. Within our corpus, first pages, last pages, and headers pose the greatest problem in this regard because in many publications, they contain the name of the author (Fig. 2). These occurrences should not be captured as mentions in order to avoid an artificial inflation of self-mentions of authors.

To solve this issue, a Python script was developed to remove from the text the headers and the names of the authors when they appeared on the first or last page of the document. This script also handled other minor cleaning tasks, such as removing page numbers and rejoining words split by line break. While the resulting textual data are still far from perfect, since the page structure could not be restored and mistakes in the OCR could not be fully repaired, they are adequately clean to minimize artifacts during mention extraction.^5^

### Identification of mentions

Once the corpus is prepared, the next step in the process is the individuation and extraction of mentions to philosophers from the texts. As we explained above, EDHIPHY focuses only on *direct mentions*, operationally defined as occurrences of proper names referring to philosophers within a document. Names occurring in bibliographic references (e.g., “Heidegger, M. (1927) *Sein und Zeit*”) are considered special cases of direct mentions.

The techniques of Named Entity Recognition developed in Natural Language Processing are the most natural candidate for extracting direct mentions (Goyal et al., 2018). Proper names referring to persons is one of the most common type of entities that these techniques are designed to recognize. Some prior studies have successfully used NER to extract names from the acknowledgments of publications in philosophy (Petrovich, 2021a) and direct mentions from scientific articles (Pence, 2022).

**Fig. 3:**
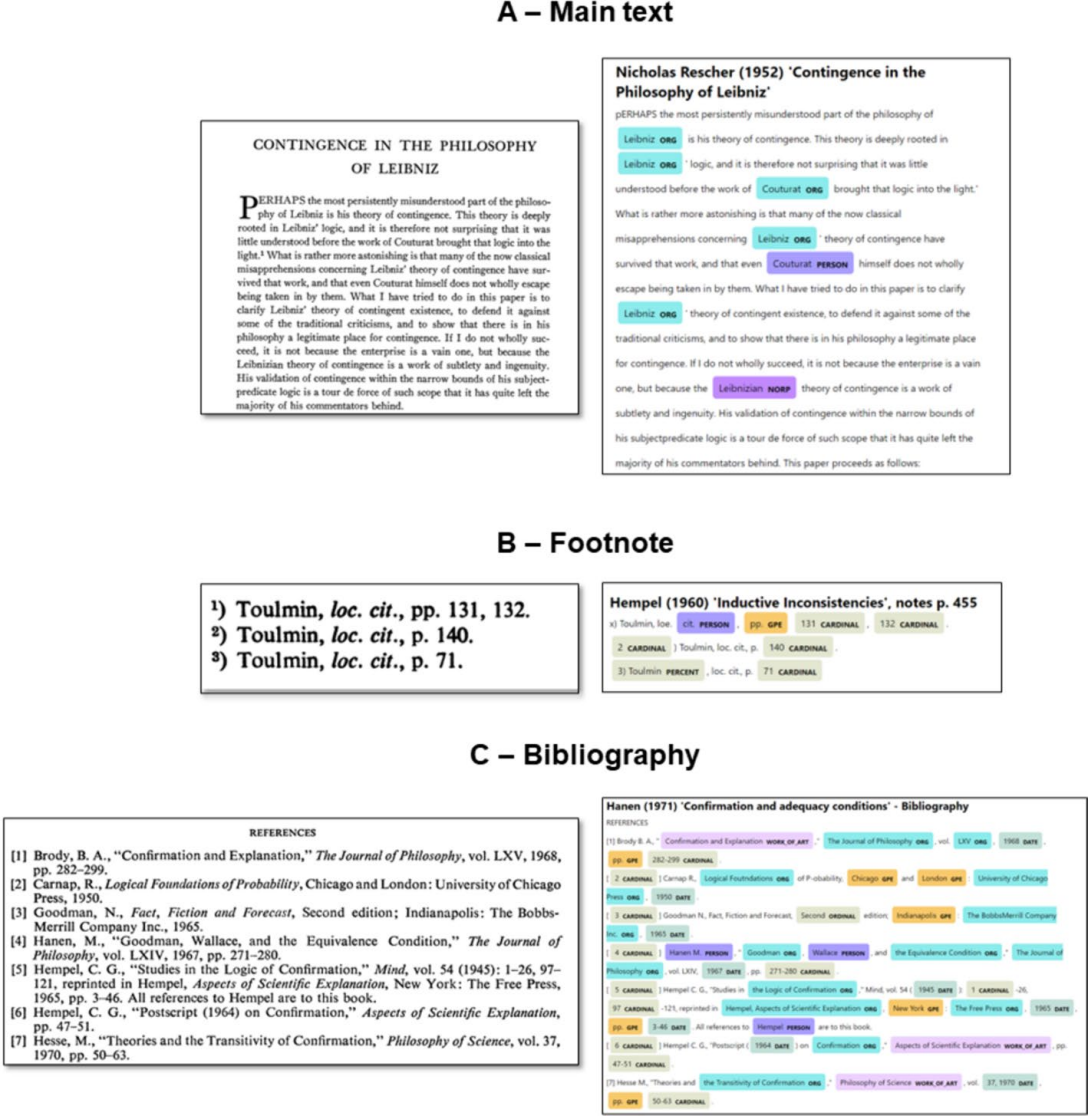
Output of spaCy NER applied to three types of texts from the corpus. The tags are visualized with spaCy visualizer (https://spacy.io/usage/visualizers)

Unfortunately, the most advanced NER systems, which rely on statistical models constructed from extensive corpora through machine learning, show very poor performance when applied to our corpus. Figure 3 shows the output of the NER module from the Python package *spaCy* on three different types of excerpts from the corpus: a fragment from the main text, a set of footnotes, and some references in a bibliography.

As these examples show, the use of machine-learning-based NER results in numerous mistakes.^6^ It incorrectly categorizes philosophers, as in Panel A where Leibniz is consistently misclassified as an organization. It shows instability in classifying the same entity, as with Louis Couturat, who is sometimes classified as a person and at other times as an organization (Panel A). Lastly, it is not able to capture mentions in para-texts, as in the case of Stephen Toulmin not being recognized as a name in footnotes (Panel B), and in Panel C, where no authors of the references are identified.

The poor performance of the NER algorithm in para-texts such as footnotes, endnotes, and bibliographies is likely due to the fact that these para-texts do not conform to standard sentence structures. The lack of a syntax hampers the parts-of-speech tagging step upon which NER relies, ultimately undermining the entire process. The disruption of sentence syntax through the artificial insertion of footnotes (see Fig. 1 above) creates similar problems in the individuation of mentions in the main text.

In theory, the performance of the NER algorithm can be improved by training it with manually annotated data.^7^ This procedure, however, is labor-intensive and time-consuming and does not guarantee that mentions in para-texts are correctly captured, due to the absence of syntax.^8^ For EDHIPHY, we adopted a different solution. Instead of relying on statistical prediction, the individuation of mentions in the text is based on a list of controlled strings, adopting a *dictionary-based approach* to NER (Goyal et al., 2018). Specifically, the NER algorithm is supplied with a dictionary, called Entity Ruler, including a predefined list of strings that the computer is instructed to extract from the texts and classify into a specific category. In EDHIPHY, these strings are names of philosophers and are classified under the new entity category ‘philosopher’. In this way, any instance of these names in the text, independently of its occurrence in the main text or in other places, will be classified as a ‘philosopher’.

While this approach might seem like a brute-force solution to the task of individuating mentions, it offers a significant advantage over the machine-learning alternative. By assigning unique identifiers to the strings incorporated into the Entity Ruler, the mentions extracted from the text can be in fact directly linked to the mentioned philosophers. In this way, dictionary-based NER simultaneously addresses the second and third tasks in constructing the mention index.

The primary drawback of using a dictionary is the loss of the ability to distinguish between different meanings of a string (Goyal et al., 2018). For instance, in the text “University of Berkeley, ” the string “Berkeley” will be extracted and categorized as a philosopher (specifically, the idealist George Berkeley, 1685–1753), even though in this context, it refers to the city in California.^9^

### Creation of the entity ruler

The creation of a list of philosophers to supply the Entity Ruler inevitably leads to the question of who qualifies as a philosopher. Any database dedicated to philosophers or philosophical works must deal with this question, and existing databases reflect varying perspectives on the nature, methods, and scope of philosophy itself (Allen & Beavers, 2011; Buckner et al., 2011). For EDHIPHY, we approached this matter pragmatically. Instead of relying on a priori definitions of ‘philosophy’ and ‘philosopher’, EDHIPHY’s list of philosophers aims to be as *comprehensive* as possible. The rationale behind this approach is that the larger the dictionary of philosophers, the greater the potential for extracting a higher number of mentions, and the lower the risk of overlooking any.

The list of philosophers used to feed the Entity Ruler is the result of merging four different datasets (Table 2). The first dataset includes all the authors of the articles in the corpus, i.e., all the authors listed in the “creator” field of JSTOR metadata (*n* = 6786 after cleaning, see the next section). Anyone who published a research article in one of the 12 journals in the corpus is therefore considered a philosopher in EDHIPHY.

**Table 2:** The four datasets that constitute EDHIPHY philosophers list. *After reconciliation and cleaning

Dataset	Source	N philosophers*
JSTOR authors	JSTOR	6786
WikiData philosophers	WikiData	32,095
American PhDs in philosophy	ProQuest	8940
Hires in top philosophy department in the US	Strassfeld (2020)	490
*EDHIPHY philosophers list*		*44,376*

Italics are used for rows of tables describing totals

The second dataset derives from WikiData, the knowledge base that serves as a central repository for structured data used by various Wikimedia projects, including Wikipedia.^10^ WikiData stores structured information about a wide range of topics, entities, and concepts in a machine-readable format (Zhao, 2023). The dataset used in EDHIPHY was retrieved from WikiData Query Service requesting all the real humans whose occupation was “philosopher”.^11^ WikiData has a great advantage in terms of inclusiveness, as it classifies as philosophers also philosophically relevant or philosophically minded scientists such as Albert Einstein, Niels Bohr or Charles Darwin, and philosophers from the non-Western world, such as Buddha and Confucius. The list of philosophers derived from WikiData is the largest of the four datasets, with 32,095 philosophers.

The source for the third dataset is ProQuest and includes the names of all people who obtained a PhD in philosophy from an American university between 1861 and 1979 ($n=8940$). Anyone with a PhD in philosophy obtained in the United States in this period is therefore considered a philosopher in EDHIPHY.

The fourth dataset originates from the data manually collected by historian Jonathan Strassfeld (Strassfeld, 2020) concerning the faculties of the eleven most prestigious philosophy departments in the United States during the mid-Twentieth century (Berkeley, Chicago, Columbia, Cornell, Harvard, Michigan, Pennsylvania, Princeton, Stanford, UCLA, and Yale). It includes 490 philosophers.

EDHIPHY’s list of philosophers is the result of the union of these four datasets properly merged.

#### Reconciliation of datasets

There is some overlap between the four datasets because several philosophers appear in more than one of the datasets. For instance, Donald Davidson appears in all four: as an author in JSTOR since he published 10 articles in the 12 journals between 1955 and 1976, as the author of a dissertation in the American PhDs list as he received his PhD from Harvard in 1949, as a professor in the Strassfeld’s list because he worked at the Universities of Chicago, Princeton and Stanford, and as an entity in WikiData as he has his own page in Wikipedia.^12^ Davidson, however, is identified by different identifiers in the datasets. In order to merge the four datasets, consequently, it was necessary to cross-reference the records and individuate the philosophers that appeared in more than one dataset. This process was complicated by the fact that, in JSTOR, no standardization of author strings is performed. The same author can thus appear under different labels: Bertrand Russell, for instance, appears in the JSTOR dataset as “B. Russell”, “Bertrand Russell”, and “B. A. W. Russell”. Hence, a correct matching of records across datasets required at the same time an extensive cleaning of data.^13^

The matching process was conducted iteratively, beginning with the merging of the JSTOR dataset and the WikiData dataset. First, each WikiData record was associated with a list of equivalent variants, which was created using the properties recorded in the WikiData entry. The list of variants included the “aliases” (i.e., the alternative names), the surname, and the first name recorded in the entry—the latter two combined in various ways to increase possible matches. Next, the JSTOR authors were compared with the WikiData entries supplied with the associated variants. When a JSTOR author matched any of the variants, it was retained as a candidate match between the JSTOR author and the associated WikiData entry. For instance, the WikiData entity “Bertrand Russell” (Q33760) was associated with the following variants: “Bertrand Russell”, “Bertrand Arthur William Russell”, “3rd Earl Russell” (derived from the WikiData aliases), “Russell” (surname recorded in WikiData), “Russell B.”, and “B. Russell” (two artificial combinations deriving from the surname and the first letter of the first name). When the JSTOR author “B. Russell” matched with the WikiData entry in virtue of the correspondence with the variant “B. Russell”, it was retained as a possible occurrence of the philosopher Bertrand Russell. Lastly, the candidate matchings were manually inspected in order to remove spurious matches and validate true matches.^14^

Note that this matching process allowed to individuate JSTOR authors that were likely to be alternative labels for the same philosopher. For instance, the JSTOR authors “Bertrand Russell” and “B. Russell” both matched with some of the variants associated with the WikiData entry Q33760 (Bertrand Russell). When different JSTOR authors matched the same WikiData entry, therefore, they were retained as candidates for merging and manually inspected to verify if the match was a true or a false positive. The matching with WikiData allowed thus to significantly improve the consistency of the JSTOR author list, individuating several alternative labels for the same philosophers.

Not every JSTOR author, however, matched a WikiData entry. In order to individuate further labels to merge, a strategy based on string similarity was applied. Specifically, the similarity between each pair of author strings was measured using Python’s *SequenceMatcher* function and clusters of similar strings were individuated using the technique of affinity propagation (Frey & Dueck, 2007). The clusters were then manually inspected to individuate true variants and discard false positives. True variants were in turn recorded as aliases of the philosopher and added to their variants list.

Next, the dataset resulting from the reconciliation of the JSTOR and WikiData datasets was combined with the Hiring and ProQuest datasets using the same approach. Again, the first round of matchings leveraged the variants associated with names. This time, the list of variants was extended by including the new aliases obtained from the JSTOR authors that had no counterpart in WikiData and further variants created by the automatic extraction of the surname.^15^ The second round was based on string similarity.^16^ All steps were followed by manual inspection in order to validate the matchings and prevent incorrect merging.

At the conclusion of this consolidation procedure, the definitive list of philosophers included into EDHIPHY comprised 44,376 philosophers. Each of them received a unique identifier enabling identification across the datasets—independently of the specific labels they had in the original source. The process allowed to clean in particular the list of JSTOR authors, reducing it from 7712 to 6786 authors, with a reduction of − 12%. Nearly half of the definitive authors were successfully matched with at least one record from another dataset (Table 3). Ultimately, 7% of EDHIPHY philosophers appear in at least two datasets, 2% in at least three datasets and 0.4% of all philosophers appear in all four datasets.

**Table 3:** Statistics on overlap among the four datasets used to generate EDHIPHY’s list of philosophers

Subset	N Philosophers	Proportion (and reference)	Notable examples
Only in WikiData	30,025	94% (of WikiData entries)	Aristotle, Plato
Only in American PhDs	7158	80% (of PhDs)	-
Only in JSTOR authors list	4145	54% (of JSTOR authors)	K. R. Srinivasa Iyengar
Only in Hires dataset	33	7% (of hires)	Bruce Kuklick
In at least two datasets	3015	7% (of philosophers in EDHIPHY)	Cora Diamond (JSTOR and WikiData)
In at least three datasets	738	2% (of philosophers in EDHIPHY)	Rudolf Carnap (WikiData, JSTOR, and Hires)
In all four datasets	181	0.4% (of philosophers in EDHIPHY)	David Lewis, Donald Davidson

#### The variants

As we saw above, the creation of the philosophers’ list makes extensive use of variants to cross-reference philosophers among the different datasets. These variants were generated in three ways: either they were directly obtained from data sources (as in the case of WikiData aliases), or they resulted from the reconciliation process (as when two JSTOR labels were recognized as referring to the same author), or they were generated by algorithms (such as artificial surnames extracted from author strings). Therefore, each philosopher in EDHIPHY is associated with a variable number of variants. Table 4 shows, for instance, those associated with Rudolf Carnap and their origin.

**Table 4:** Variants associated with Rudolf Carnap (P:5858) and their origin. *Variant created combining the WikiData surname with the initial of the WikiData first name

Variant	Origin
Rudolf Carnap	JSTOR label
R. Carnap	JSTOR label
Rudolph Carnap	WikiData alias
Carnap	WikiData surname
Carnap R.	Artificial variant*

EDHIPHY includes a total of 139,623 variants obtained in 13 different ways. The most common variants (24% of the total) are those generated algorithmically by combining the initial of the first name with the surname (e.g., “Carnap R.”).^17^ The mean number of variants per philosopher is 3.7 (median = 4, standard deviation = 2.3).

Incorporating variants into the dictionary of the Entity Ruler significantly improves Named Entity Recognition’s *recall*, as these variants expand the range of patterns that the computer is instructed to extract from the text and recognize as mentions to philosophers.

The Entity Ruler allowed the extraction of 1,095,765 mentions from EDHIPHY’s corpus. Of all the articles, 98% include at least one mention. When we consider only those articles, the average number of mentions per article is 41.1 (median = 26 mentions, standard deviation = 53.7 mentions). The distribution of mentions ends up being right-skewed, with a tail of articles containing a high number of mentions.

### Linking mentions to philosophers

Once the full-texts have been properly cleaned and mentions have been extracted from the full-texts leveraging the Entity Ruler, the third crucial step in the creation of the mention index is *linking* the extracted mentions to the philosophers they are referring to. This step is equivalent to the establishment of citation links in the context of a citation index.

If it was possible to establish a one-to-one correspondences between mentions and philosophers, as it happens between a document and its cited references in a citation index, this process would be straightforward. Unfortunately, many mentions extracted from the corpus break the one-to-one correspondence in two ways. On the one hand, there are mentions that are linked to philosophers by a *many-to-one* relationship. This happens when the same philosopher is mentioned in different ways. Aristotle, for instance, can be mentioned as “Aristotle”, “Aristoteles”, “Aristotelis”, or even “the Stagirite”. These many-to-one relationships represent cases of *synonymity* of mentions. On the other hand, there are mentions that are linked to philosophers by a *one-to-many* relationship. This happens when different philosophers are mentioned in the same way. For instance, the mention “Kuhn” can refer both to the philosopher of science Thomas Kuhn and to the logician Steven Kuhn. These one-to-many relationships represent cases of *homonymity* of mentions. By contrast, mentions that are characterized by one-to-one correspondence with philosophers, i.e. mentions that have neither synonyms or homonyms, represent *univocal* mentions. For instance, the mentions “Kant” and “Hegel” in EDHIPHY are univocal because they can refer only to Immanuel Kant and Georg W. F. Hegel, respectively.

The variants associated with each philosopher in EDHIPHY allow to successfully resolve the issue of synonymity as they allow to attribute different extracted mentions to the same philosopher. For instance, any occurrence of the variants listed in Table 4 above will be attributed to Rudolf Carnap.

Variants, however, do not resolve the issue of homonymity. In fact, they make it even more difficult to solve, to the extent that a consistent number of variants result to be associated with more than one philosopher.^18^ The variant “Russell”, for instance, is shared by 10 different philosophers in EDHIPHY, in addition to the famous British philosopher Bertrand Russell, because it is a common surname. Similarly, “Marx” is shared by 3 additional philosophers, besides Karl Marx. In EDHIPHY, 12% of the variants are *ambiguous* in this sense. Although their weight in the set of variants is relatively negligible, their impact on the linking of mentions is significant. In the end, 41% of mentions (i.e., more than 450,000 mentions) extracted from the corpus turn out to be ambiguous. This disproportionate percentage is due to the fact that some highly mentioned philosophers, such as Russell and Marx, are associated with ambiguous variants. Finding a strategy to disambiguate the ambiguous mentions, that is, to link them to the philosopher they refer to, is therefore a necessary step in the creation of the mention index, in order to make it an effective research tool.

#### Disambiguation of mentions

The first step in the disambiguation process was to associate all the ambiguous mentions with the set of philosophers they could potentially refer to. In the following, we will call the members of this set the “alternatives” associated with a mention. For instance, a mention phrased like “Russell” was associated with all 11 philosophers who share the variant “Russell”, as any of them could be the intended referent for the mention. Disambiguating ambiguous mentions consists of identifying the philosopher that was actually referred to in the text, selecting from the alternatives associated with the mention.

The disambiguation method used in EDHIPHY relies on four different strategies that proceed in a logical progression from micro (the individual article) to macro (the entire corpus). The strategies are applied sequentially, meaning that mentions not disambiguated through the first strategy are addressed by the second, those remaining after the second strategy by the third, and so forth.

The first strategy leverages the univocal mentions found in the same article where the ambiguous mention occurs. The basic idea behind this strategy is that authors mention univocally the philosophers at least one time in their articles, in order to help the reader understand who they are mentioning. For instance, we expect that an author referring to Bertrand Russell will use a univocal mention (like “Bertrand Russell”) at least one time in the article, before resorting to ambiguous mentions (like the simple “Russell”). In this way, the reader will have no uncertainty about which Russell is being referred to among the various philosophers with the name Russell. Indeed, using the univocal mentions that occur in an article to disambiguate the ambiguous ones is the same strategy that a human reader would apply in order to figure out the identities of the philosophers that are mentioned in an article. In EDHIPHY, this strategy is implemented as follows. All the mentions extracted from an article are divided into two sets: the univocal and the ambiguous. The set of philosophers surely mentioned in the article is derived from the set of univocal mentions. Next, the set of alternatives associated with each ambiguous mention is compared with the set of univocally mentioned philosophers: if the intersection between the two contains only one philosopher, then the ambiguous mention is disambiguated and its referent is individuated in the alternative belonging to the intersection. For instance, let ${U}_{a}=\left\{{P}_{1},{P}_{2},{P}_{3}\right\}$ be the set of philosophers univocally mentioned in article $a$, and ${A}_{m}=\{{P}_{1},{P}_{5},{P}_{6}, {P}_{7}\}$ the set of alternatives associated with the ambiguous mention $m$ occurring in the same article $a$. Since ${U}_{a}\cap {A}_{m}\equiv \{{P}_{1}\}$ and $\left|{U}_{a}\cap {A}_{m}\right|=1$, the mention $m$ will be successfully disambiguated and linked to philosopher ${P}_{1}$. By contrast, when the intersection between the two sets is either empty or contains more than one element, the disambiguation fails and the mention’s status is left ambiguous. This strategy enabled the disambiguation of 14.8% of the mentions extracted from the corpus.

The second strategy is conceptually identical to the first, but leverages as reference context all the articles produced by an author, i.e., the *oeuvre*, instead of the individual article.^19^ Similarly to the first strategy, the set of philosophers univocally mentioned within the *oeuvre* of an author is compared with the set of alternatives associated with each ambiguous mentions. When the intersection between the sets yields only one element, the ambiguous mentions is disambiguated and linked to the philosopher belonging to the intersection. Otherwise, the disambiguation process fails. Through this second strategy, 3.5% of mentions were disambiguated.

These two strategies can be thought of as a way to equip the algorithm with a *memory* resembling that of a human reader. The first strategy results in a memory for the philosophers univocally mentioned in an article, which can be used to identify the intended referents of the ambiguous mentions within the same article. The second strategy extends this memory to the entire corpus of an author’s articles, simulating the way a human reader might browse through an author’s works to figure out the referent of an ambiguous mention discovered anywhere within those articles.

The third and fourth strategy, by contrast, reflect to a lesser extent how a human reader would address the task of disambiguation. They are algorithmic operationalizations of heuristics that might be used by humans, though.

The third strategy relies on *mention counting* all over the corpus—something that a computer, differently from a human, can easily do. In a first step, the number of mentions that each philosopher univocally mentioned in the corpus receives in the whole corpus is computed.^20^ Then, all the alternatives that appear in the sets associated with ambiguous mentions and that collect 0 mentions in the corpus are *excluded* from the alternative sets of ambiguous mentions. If the set remains with only one element, the ambiguous mention is disambiguated and linked to the philosopher that survived the process of exclusion. By contrast, if more than one philosopher remains, the disambiguation fails. For instance, let ${A}_{m}=\left\{{P}_{8},{P}_{9},{P}_{10}\right\}$ be the set of alternatives associated with the ambiguous mention $m$ and let say that ${P}_{8}$ receives 10 mentions in the corpus while ${P}_{9}$ and ${P}_{10}$ are never mentioned. Then, mention $m$ is successfully disambiguated and linked to ${P}_{8}$. This strategy mimics a heuristic based on reputation, as it favors the philosopher who is more “famous” in the corpus as the solution to the disambiguation. It allowed to disambiguate 5.2% of mentions.

The fourth strategy leverages co-mention frequencies. In a first step, the co-mention matrix $C$ including all the philosophers univocally mentioned in the corpus is computed. Each element ${c}_{i,j}$ of the matrix represents how many times philosopher $i$ is co-mentioned with philosopher $j$.^21^ In this way, each column $\overrightarrow{{P}_{i}}$ of this matrix represents the *co-mention vector* of a philosopher, which encodes how many time that philosopher is co-mentioned with all the other philosophers mentioned in the corpus. As expected, the co-mention matrix $C$ is highly sparse, as around 99% of its elements are 0 s. In the second step, the *mention vectors* of articles where ambiguous mentions occur are computed. These vectors encode in their components how many times the (univocally mentioned) philosophers are mentioned within these articles. For instance, if articles $a$ mentions ${P}_{1}$ 10 times, ${P}_{2}$ 5 times and ${P}_{3}$ 2 times, the mention vector associated to $a$ will be $\overrightarrow{a}=\langle 10, 5, 2\rangle$. In the last step, the co-mention vectors of all philosophers associated with an ambiguous mention in a certain article are compared with the mention vector of the articles using the *cosine similarity*:

$$S_{c} \left( {A,B} \right) = \cos \left( \theta \right) = \frac{{\vec{P} \cdot \vec{a}}}{{\left\| {\vec{P}} \right\|\left\| {\vec{a}} \right\|}} = \frac{{\sum\nolimits_{{i = 1}}^{n} {P_{i} } a_{i} }}{{\sqrt {\sum\nolimits_{{i = 1}}^{n} {P_{i}^{2} } } \sqrt {\sum\nolimits_{{i = 1}}^{n} {a_{i}^{2} } } }}$$

where $\overrightarrow{P}$ and $\overrightarrow{a}$ are the co-mention vector of the philosopher and the mention vector of the article, respectively, and ${P}_{i}$ and ${a}_{i}$ are their $i$-th component.^22^ The philosopher whose co-mention vector shows the highest similarity with the mention vector of the mentioning article, i.e. the philosopher whose co-mention vector shows the smaller angle with the articles’ mention vector, is thus selected as solution to the disambiguation. For example, let us say that ${A}_{m}=\{{P}_{8},{P}_{9}\}$ is the set of philosophers associated with the ambiguous mention $m$ occurring in article $r$, $\overrightarrow{{P}_{8}}$ and $\overrightarrow{{P}_{9}}$ are the co-mention vectors associated with the alternatives ${P}_{8}$ and ${P}_{9}$, and $\overrightarrow{r}$ is the mention vector of article $r$. Let us further say that ${S}_{C}\left(\overrightarrow{r}, \overrightarrow{{P}_{8}}\right)=0.8$ and ${S}_{C}\left(\overrightarrow{r}, \overrightarrow{{P}_{9}}\right)=0.2$, where ${S}_{C}$ is the cosine similarity between vectors. Then, the solution to the disambiguation of $m$ will be ${P}_{8}$ because it is the philosopher whose co-mention vector is closer to the mention vector of article $r$.

Cosine similarity was chosen over alternative similarity measures because it is characterized by the desirable property of being insensitive to the magnitude of vectors, focusing solely on the angle between them (Jones & Furnas, 1987; Salton & McGill, 1983). This characteristic is particularly crucial in our scenario, where the magnitude of co-mention vector components is frequently significantly greater than that of mention vector components.

Moreover, differently from the previous three strategies, the fourth strategy can be fine-tuned by two parameters, $\alpha$ and $\beta$. $\alpha$ specifies a minimum threshold of similarity between the mention and co-mention vectors to accept a disambiguation solution. This threshold permits to exclude those disambiguation solutions that, even if result to be the ones with the highest similarity among the alternatives, show nonetheless a too low absolute value of similarity with the article mention vector. In EDHIPHY, the value of $\alpha$ was set to 0.01. The parameter $\beta$, by contrast, specifies the minimum threshold of *relative* difference between the ordered similarities of alternatives. This parameter serves to exclude those disambiguation solutions where the alternatives with the highest similarity with the mention vectors show in fact a too small difference and, thus, are all plausible solutions to the disambiguation. In EDHIPHY, the value of $\beta$ was set to 0.25, meaning that the similarity of the second-rank alternative should be at least 25% less than the similarity of the first-rank alternative. The rate of disambiguation of the fourth strategy depends on the settings of $\alpha$ and $\beta$. Low values of the two parameters increase the number of disambiguated mentions at the cost of a higher probability of erroneous disambiguation. Conversely, high values decrease the number of disambiguated mentions but increase the probability of correct attributions. The fourth strategy with the settings of $\alpha$ and $\beta$ indicated above, allowed to disambiguate 10.8% of the mentions.

Leaving aside technical details, this fourth strategy attempts to simulate an heuristic based on the principle “Tell me who you go with and I’ll tell you who you are”. A real example from EDHIPHY will help clarifying the underlying idea. Let us say that a certain article in the corpus mentions a philosopher named “Sellars”. “Sellars”, however, may refer to either Roy Wood Sellars (1880–1973) or Wilfrid Sellars (1912–1989), respectively father and son and both prominent American philosophers. Let us imagine that the article never gives the first name of the Sellar it is referring to, so that we are left uncertain on who is the intended Sellars.^23^ One way to figure out the referent is to examine the other philosophers that are mentioned in the article. Let us say that the article mentions, in addition to the ambiguous Sellars, also Alfred N. Whitehead, George Santayana, and John Dewey. These are philosophers with whom Roy Wood Sellars frequently engaged in discussions and who were part of the same intellectual *milieu* as him. Wilfrid Sellars, by contrast, belonged to a different epoch of American philosophy, dominated by analytic philosophers such as W. V. O. Quine and Rudolf Carnap (and Wilfrid Sellars himself). Combining the information about the philosophers mentioned in the article with historical information about the philosophers intellectually close to the two Sellars, therefore, we can plausibly conclude that the Sellars mentioned in the article is in fact Roy Wood and not Wilfrid Sellars. The fourth disambiguation strategy encodes the intellectual closeness between philosophers in the co-mention matrix and leverages this information to choose the philosophers who is “closest” to the article’s intellectual profile.

At the end of the disambiguation process, only 7% of the mentions ($n=\text{77,192}$) remained ambiguous. This means that 93% of mentions extracted from the corpus are linked to a specific philosopher.

**Fig. 4:**
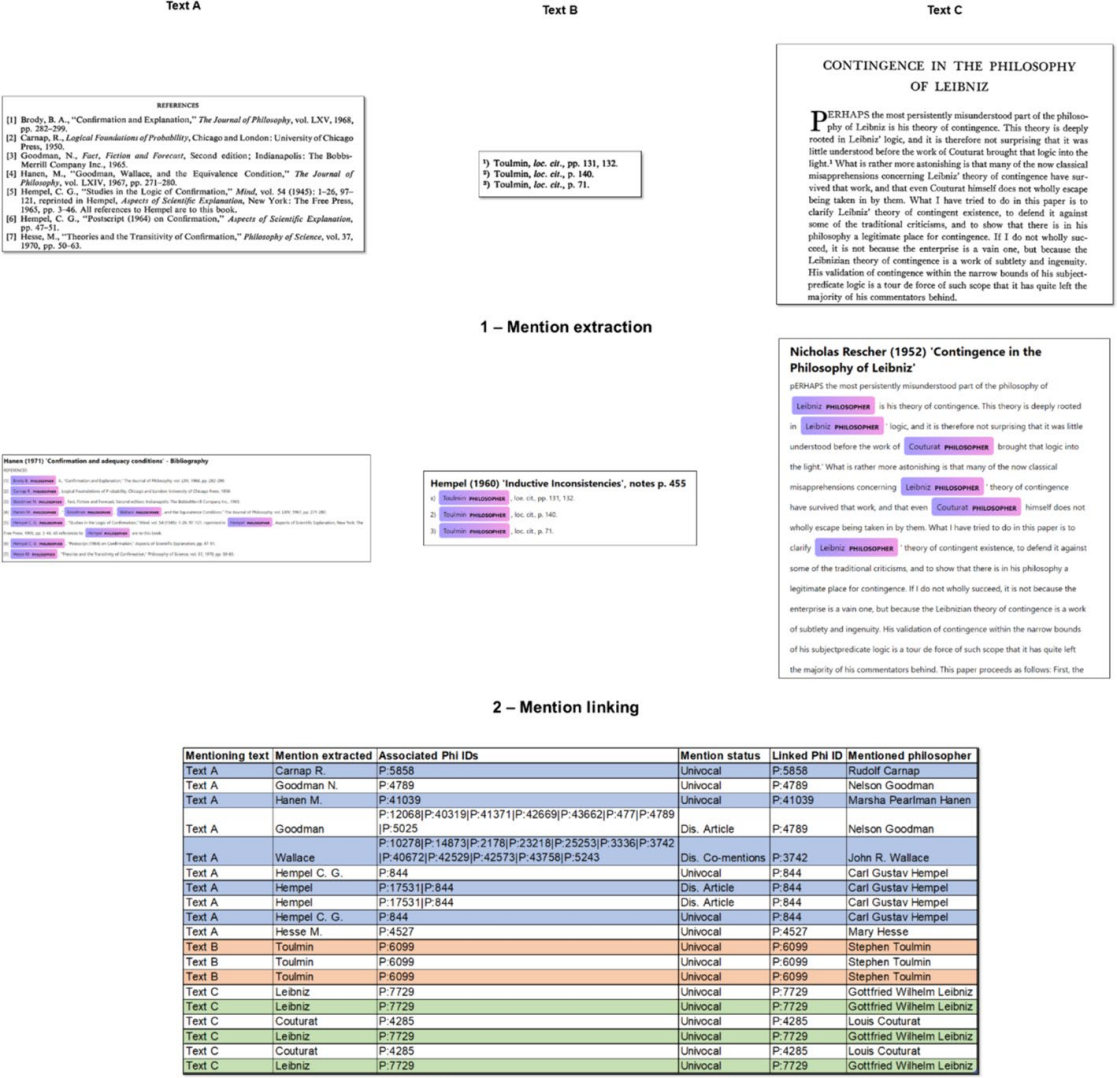
Processing of texts in EDHIPHY from mention extraction to mention linking. Alternatives are separated by “|” in the column Associated Phi IDs

Figure 4 shows how the same three excerpts appearing in Fig. 3 above are processed in EDHIPHY. In the first step, mentions are extracted from the text using the NER supplied with the Entity Ruler. In the second step, mentions are linked to the philosopher they refer to. If required, a disambiguation strategy is employed to select the most likely referent among the alternatives.

We acknowledge that for corpora from other research fields, geographic areas, cultural traditions, or periods, our disambiguation strategies could be less effective. Corpora characterized by widespread homonymity, such as Chinese or Korean corpora, are likely to require additional disambiguation steps or even significantly different approaches. For instance, the disambiguation process could consider additional properties of the alternatives associated with an ambiguous mention, in addition to mentions and co-mentions. These properties might include topical similarity between the article and the intellectual profile of the alternatives, or the temporal distance between the publication year and the birth year of the alternatives. Relevant insights in this regard can be found in the literature on author name disambiguation (see the recent review by Rodrigues et al., 2024).

#### Improving mention linking

The performance of the mention linking process in EDHIPHY can be significantly improved by rectifying the inevitable errors that occur during mention extraction and disambiguation. EDHIPHY is thus equipped with ad-hoc cleaning solutions and cleaning files designed to track and rectify these errors.

The first ad-hoc cleaning solution involves removing from the Entity Ruler all variants associated with philosophers born after 1960 ($n=\text{15,010}$, 11% of all variants, see Table 5).^24^ Given the corpus’s timespan, which does not include publications after 1979, it is highly unlikely to encounter mentions to these philosophers. By excluding these variants, the number of alternatives associated with ambiguous variants is reduced, simplifying the disambiguation process and enhancing its reliability.

**Table 5:** Incidence of valid and problematic variants in EDHIPHY

Issue	Variants	Perc
No issue (valid variants actually used in the Entity Ruler)	123,296	88%
Variant associated with a too young philosopher	15,010	11%
Too short variant	856	0.6%
Homonym variants	415	0.3%
Unlikely variant	46	0.03%
*Total*	*139,623*	*100%*

Italics are used for rows of tables describing totals

Similarly, the second ad-hoc cleaning solution consists of removing from the Entity Ruler all variants that are shorter than four characters ($n=856$, 0.6% of all variants). Again, the exclusion is motivated by the high rates of false positives that these variants produce. Unfortunately, this move does come at a cost, as it excludes variants, such as “Pap”, “Coe”, or “Eco”, which are associated with fairly well-known philosophers (respectively, Arthur Pap, George Coe, and Umberto Eco) or variants such as “Qi” or “Zhy”, which are associated with several Chinese philosophers.

The cleaning files consist of two lists of variants. The first list comprises variants that, in most cases, yield *false positives*. The second list includes variants that, in most cases, lead to *erroneous mention attributions*, i.e., to errors in the mention linking process. Both lists were compiled through manual inspection of the results of mention extraction and iteratively refined.^25^

The list of variants inducing false positives includes variants that coincide with proper names having multiple meanings. For instance, the variant “Paris”, which is one of the variants associated with the French philosopher Edmond Paris (1894–1970), generates false matches whenever the city of Paris is mentioned, artificially inflating mentions linked to Edmond Paris. Additional examples of problematic variants of this kind include “Caesar”, “England”, “Even, ” “Saint”, and “Springer”. As noted above, this drawback arises from the dictionary-based approach to NER adopted in EDHIPHY. Individuating these problematic variants permits, however, their exclusion from the Entity Ruler and significantly reduces the incidence of false positives.

The list of variants that induce errors in the mention linking process, on the other hand, includes variants that introduce noise into the disambiguation process, particularly affecting disambiguation based on co-mentions. These variants typically belong to relatively minor philosophers who happen to share a variant, usually the surname, with a highly well-known philosopher. For instance, the American philosopher and educator Alain LeRoy Locke (1885–1954) shares the same surname as John Locke, one of the most influential of Enlightenment philosophers. Due to John Locke’s immense influence on Western philosophy, many authors in the corpus simply refer to him as “Locke”. However, from the Entity Ruler’s perspective, these mentions are ambiguous because they match the variant “Locke”, which is shared between John Locke and Alain LeRoy Locke. When the disambiguation of “Locke” cannot be solved through the first three strategies, the fourth strategy, based on co-mentions, sometimes incorrectly links “Locke” to Alain LeRoy Locke, artificially inflating mentions attributed to him. To prevent this type of errors, the second list of problematic variants allow to flag some variants as “unlikely”, excluding them from the disambiguation process. Note, however, that this list is relatively short, containing fewer than 50 variants, reflecting its limited role in the disambiguation process.

Table 5 shows the incidence of the different types of variants in EDHIPHY.

**Fig. 5:**
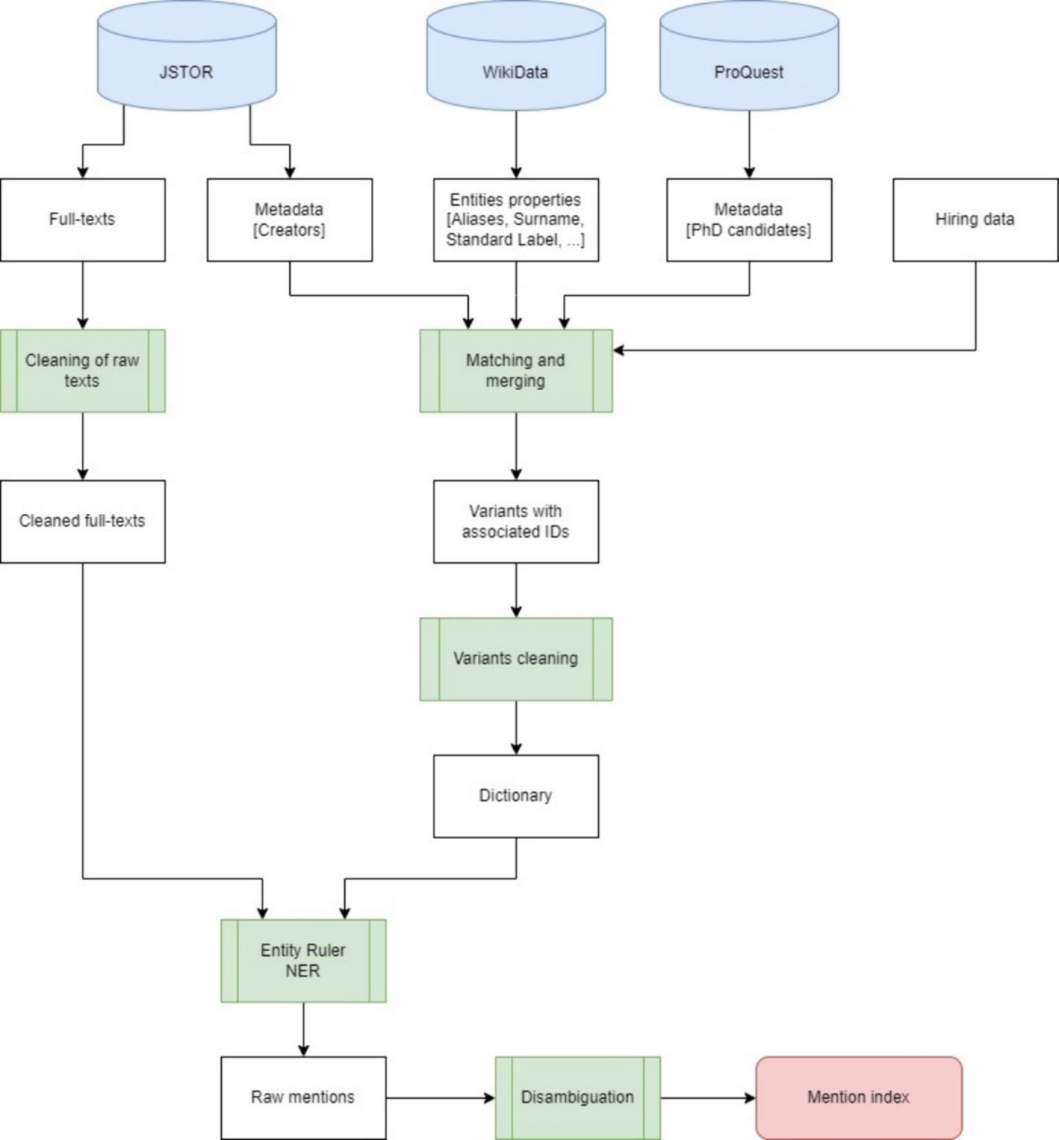
Diagram summarizing the creation of the mention index

Finally, the diagram in Fig. 5 summarizes the entire procedure that leads to the creation of the mention links in EDHIPHY, starting from the data sources and ending with the mentions linked to the philosophers, i.e., the mention index.

#### Evaluation of linking

Both mention extraction and mention linking are error-prone processes. Even if they can be improved by ad-hoc solutions such as those described in the previous section, it is important to have a quantitative measure of their accuracy, especially in terms of *precision* (proportion of correct links over the total number of links). Note that the precision of commercial citation databases is not always transparently declared: Web of Science average missed citation rate has been estimated to be between 5 and 12% (Olensky et al., 2016; van Eck & Waltman, 2019).^26^

**Fig. 6:**
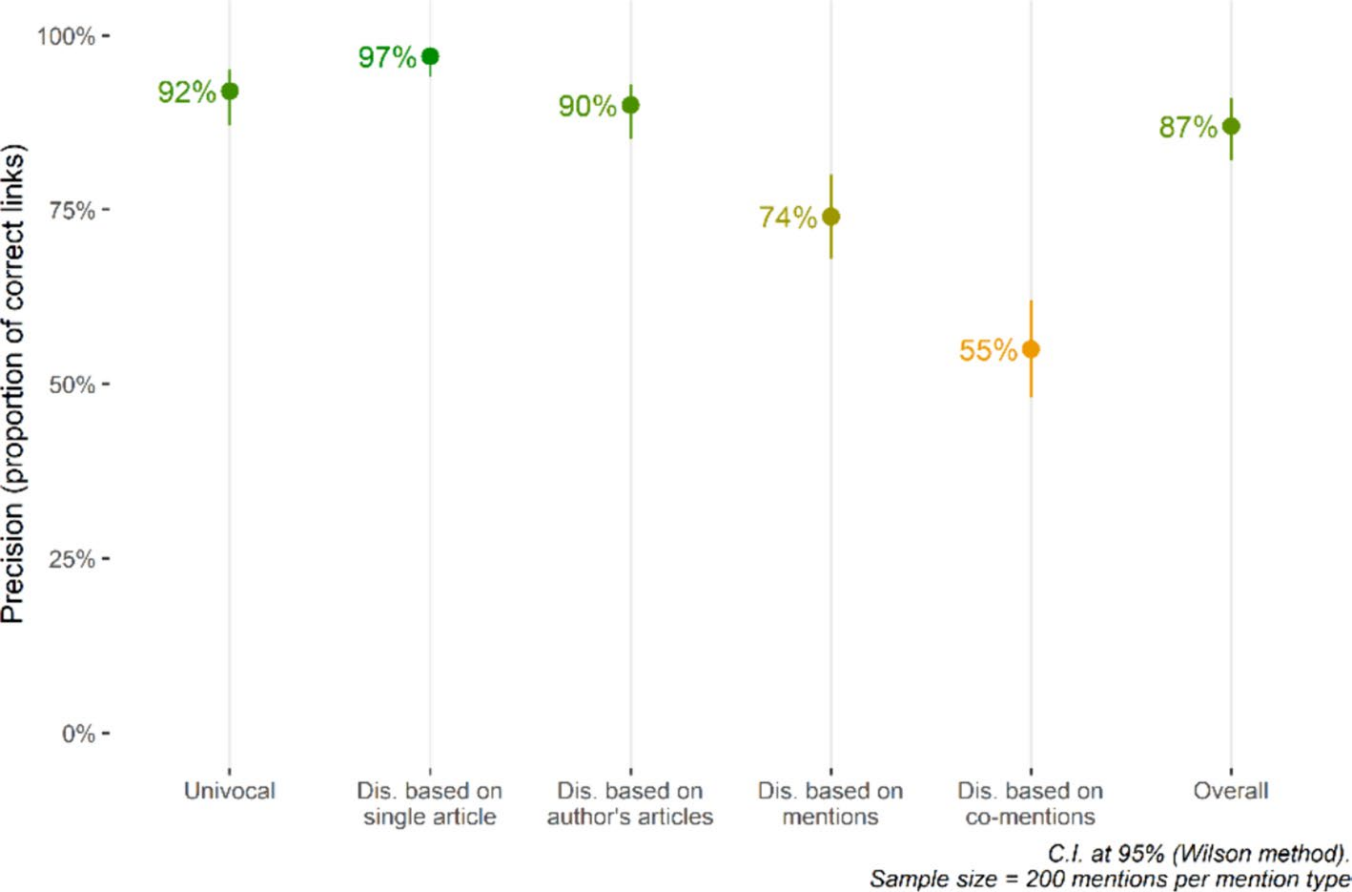
Precision estimates broken down by mention type. Error bars represent the confidence interval around the observed value

To evaluate the reliability of the mention links, we randomly extracted 200 mentions for each type of mention in EDHIPHY (univocal mentions, mentions disambiguated based on the individual article, mentions disambiguated based on author’s articles, mentions disambiguated based on mentions, and mentions disambiguated based on co-mentions). Then, we manually checked whether the philosopher mentioned in the article coincided with the philosopher indicated in EDHIPHY or not. Table 6 and Fig. 6 below summarize the results of the assessment.

**Table 6:** Evaluation of EDHIPHY’s precision in linking mentions. Sample size = 200 mentions per type. *95% Confidence Interval computed with Wilson method

Type of mentions	Number	Incidence	Sample precision	Estimated precision*
Univocal	643,281	59%	92%	87%–95%
Disambiguated based on individual article	162,265	15%	97%	94%–98%
Disambiguated based on author’s articles	38,439	4%	90%	85%–93%
Disambiguated based on mentions	56,624	5%	74%	68%–80%
Disambiguated based on co-mentions	117,964	11%	55%	48%–62%
Ambiguous	77,192	7%	-	-
*Total*	*1,095,765*		*87%*	*82%–91%*

Italics are used for rows of tables describing totals

The overall precision of EDHIPHY is adequately high, with 82%-91% of mentions linked to the correct philosopher. As expected, the four disambiguation strategies exhibit varying precision levels. The strategies that rely on information closely related to the ambiguous mentions (the first and second strategies) exhibit higher precision compared to the strategies that rely on information derived from the entire corpus (the third and fourth strategies).

Errors in the linking of mentions occur in a variety of situations and for diverse reasons. The most common scenarios involve strings erroneously recognized as philosophers but actually referring to something else (a characters, a place, a publisher, a concept, etc.) and cases in which individuals who are not included in EDHIPHY’s list of philosophers are mentioned in articles and they happen to share a variant with a philosopher who is on the list.

A typical example of the first situation involves fictional characters sharing names with real philosophers. For example, in the article ‘Kirk on Quine on Bilingualism’ (http://www.jstor.org/stable/2252628), two characters named Aman and Beeman appear in a thought experiment. The character Aman coincidentally has the same surname as Kenneth J. Aman (1937–1998). Because of this homonymity, mentions of the fictional character Aman are erroneously linked to Kenneth Aman. Another related mistake occurs when mentions of biblical characters, like Adam, are incorrectly linked to philosophers whose surname coincides with the biblical name (e.g., Charles Adam). Another example of these errors includes place names that are mistaken for philosophers because they happen to coincide with their surname. For instance, in the article ‘Mental Copies’ (http://www.jstor.org/stable/2183811), “White” in “White House” is incorrectly interpreted as a mention of Morton White (1917–2016). In rare cases, even concepts can be misidentified as philosophers when they are written with a capital letter. For instance, in the article ‘Descartes and Modern Theories of Emotion’ (http://www.jstor.org/stable/2175567), the emotion of “Hope” is wrongly recognized as the philosopher Richard Hope.

Errors of the second type occur when an author mentions artists, scientists, or individuals who are not covered by the Entity Ruler, and these individuals happen to have names (or parts of their names) that overlap with a philosopher listed in the Entity Ruler. For instance, in the article ‘Space, Time and Falsifiability’ (http://www.jstor.org/stable/186137), reference number 74 refers to a book written by the physicist John A. Wheeler, who is not included in EDHIPHY’s list of philosophers. The surname “Wheeler”, however, is a univocal variant associated with James T. Wheeler (1824–1897). Consequently, the mention of John A. Wheeler is wrongly attributed to James T. Wheeler. Likewise, mentions to the mathematician John Von Neumann, who is not included in EDHIPHY, are attributed to Michael Neumann (1946-) because the “Neumann” in “Von Neumann” matches the philosopher’s surname.

These errors show that certain philosophers are more likely than others to elicit erroneous linking, namely the philosophers whose variants coincide with some proper name and those with relatively common surnames. This means that the level of precision of mention linking is not uniformly distributed among EDHIPHY’s philosophers. For philosophers with very distinctive variants, such as Kant, Hegel, Heidegger or Wittgenstein, the level of precision of the mention linking process is consistently higher than for philosophers with common names, such as William James or John Brown. The latter type of philosophers are more likely than the former to receive false mentions. This varying level of precision should be taken into account when interpreting mention statistics produced with EDHIPHY.

### Enriching the mention index

The four datasets used to compile EDHIPHY’s list of philosophers contain a wealth of data beyond just the philosophers’ names. These additional data have been leveraged to enhance the mention index and significantly expand the types of analyses that EDHIPHY can support.

First, all the metadata from JSTOR articles have been integrated into EDHIPHY. This integration enables the breakdown of mentions along various dimensions, such as the journal or the publication year of the articles in which they occur. Most importantly, standardizing JSTOR authors and including them in the philosophers’ list allows the creation of a consistent *mention network*, where philosophers can appear as both generators of mentions (when they author articles in the corpus) and receivers of mentions (when they are mentioned within the corpus articles). Such a mention network enables the computation of numerous statistics and network centrality measures.

Furthermore, the philosophers themselves can be linked to various properties derived from the four datasets. Philosophers recorded in WikiData are associated with gender and birth year information. Those found in the ProQuest dataset are linked to PhD dissertations that include title, earning year, and granting institution details. For philosophers listed in Strassfeld’s dataset, comprehensive career information spanning from 1930 to 1979 is available, including institutional affiliations, ranks, and the years they began and ended their positions. Strassfeld also classified philosophers depending on their philosophical approach (historical vs analytic). Depending on how many datasets a philosopher appears in, these diverse properties become accessible for analysis.

Lastly, mentions themselves are associated with their mention context, i.e., the portion of text that surrounds them in the mentioning article. Mention contexts, similarly to citation contexts, allow potentially to further characterize mentions, for instance through sentiment analysis (Sula & Miller, 2014) or epistemological analysis (Petrovich, 2018).

The full relationship diagram of EDHIPHY, reported in the Online Supplementary Materials, shows how the various properties of the entities appearing in EDHIPHY relate to each other.

We highlight that, thanks to its modular structure, EDHIPHY can easily integrate additional data and features, both for articles and philosophers. A natural extension could involve applying advanced NLP techniques to the corpus of full texts. For instance, phrase mining (Cheng et al., 2023) and topic-modeling (Malaterre & Lareau, 2022) could be used to associate each article with a set of keywords and a topic distribution, which could then be related to the mentioned philosophers. This would allow for tracing philosopher/concept or philosopher/topic pairs at a fine-grained level. Methodologically, this information could even be used to improve the disambiguation process (see Sect. “Disambiguation of mentions” above).

The next section presents several applications of EDHIPHY to illustrate how it can be used as an effective research tool to answer questions about the structure and development of Anglophone philosophy in the twentieth century, specifically the impact of intellectual migration on the development American philosophy in the years after World War II.

## EDHIPHY and mention analysis in action

When fascist regimes rose to power in Central-Europe in the 1930s, this created a massive wave of (intellectual) migration, which shifted scientific and cultural activities across the globe (Palmier, 2006). This shift was responsible for the most important development in twentieth century philosophy, namely the rise of a fierce intellectual divide between so-called analytic philosophy, practiced mostly in Anglophone countries, and continental philosophy, practiced in Continental Europe (Friedman, 1999). It is generally assumed that the rise of an analytic style in American philosophy was at least partially caused by the successful migration of the logical empiricist movement from the German-speaking world to the USA (Hardcastle & Richardson, 2006). From the late 1930s onward, logical empiricist philosophers not only succeeded to influence upcoming younger American philosophers, like Ernest Nagel, Nelson Goodman, or W. V. Quine, but also managed to become important philosophers at key institutions, e.g. Hans Reichenbach at UCLA, Rudolf Carnap at Chicago and Carl Hempel at Princeton and Yale (2020b; Verhaegh, 2020a). Even though the USA also welcomed many proponents of other (mostly German) philosophical schools such as Neo-Kantianism, critical theory, and phenomenology, these intellectual migrants never succeeded in driving the philosophical research agenda in the USA (Strassfeld, 2022; Wheatland, 2009).

Until now, historical research on the impact of migration on twentieth century philosophy was limited mostly to qualitative studies of archival sources and interpretive readings of key publications. EDHIPHY creates the possibility to do a broader analysis of the impact of migration which takes the entire publication record of professional American philosophy journals into account. This type of research cannot be done using standard citation analyses since (1) very few articles from this period include citations or list of references and (2) these publications predate the period covered by existing citation databases. Below, we briefly discuss four types of analyses to illustrate how the mention index and EDHIPHY can be used to strengthen the empirical base for historical research and offer new avenues for explanatory exploration.

### Quantity of mentions as proxy for intellectual success

If the traditional histories of philosophical migration in the twentieth century are correct, we would expect that logical empiricist refugees by the 1950s were more successful academically than refugees from other schools. Using mentions as a proxy for academic success, the data from EDHIPHY can illustrate this discrepancy quantitatively. Table 7 shows the top 50 most-mentioned philosophers in American journals in the 1950s. To limit our analysis to the reception of contemporaries, we have constrained the table to mentions to philosophers born after 1850.^27^

**Table 7:** Top 50 most-mentioned philosophers in six American journals 1951–1960. Mentions of philosophers marked with an asterisk (*) are likely to be false positives due to the homonymity of their surnames (see Sect. “Evaluation of linking” above). We decided to keep them in here to indicate the relatively low frequency of false positives

Rank	Philosopher	Mentioning articles	Rank	Philosopher	Mentioning articles
1	John Dewey	290	26	Nelson Goodman	74
2	Bertrand Russell	286	27	Ernest Nagel	67
3	Rudolf Carnap	258	28	Edwin Holt	66
4	Alfred N. Whitehead	209	29	P. F. Strawson	62
5	Willard V. O. Quine	168	30	Ernst Cassirer	58
6	G. E. Moore	167	31	Ullin Place	58*
7	Clarence I. Lewis	163	32	Jean-Paul Sartre	58
8	Ludwig Wittgenstein	161	33	Henry Margenau	55
9	Albert Einstein	141	34	Arthur Prior	55
10	A. J. Ayer	138	35	Alfred Tarski	53
11	Herbert Feigl	130	36	John Maynard Keynes	53
12	Henri Bergson	119	37	George Herbert Mead	51
13	Hans Reichenbach	114	38	John Wisdom	51
14	Gilbert Ryle	111	39	Roderick Chisholm	51
15	Wilfrid Sellars	111	40	Henry George	51*
16	C. D. Broad	109	41	Philip Scribner	50*
17	George Santayana	107	42	Josiah Royce	49
18	Max Black	105	43	Sidney Hook	48
19	Edmund Husserl	100	44	H. H. Price	47
20	Carl G. Hempel	89	45	Karl Popper	47
21	Alonzo Church	86	46	Moritz Schlick	47
22	Charles L. Stevenson	86	47	Niels Bohr	45
23	Martin Heidegger	82	48	John Hospers	44
24	Paul A. Schilpp	77	49	Stephen Toulmin	44
25	William D. Ross	76	50	Desmond Henry	43*

This table illustrates the extent to which migrating philosophers from schools of thought other than logical empiricism did not find much reception in professional American philosophy in the 1950s. Five members of the logical empiricist movement who migrated to the USA are in the top 50—Carnap (nr. 3), Feigl (nr. 11), Reichenbach (nr. 13), Hempel (nr. 20), and Tarski (nr. 35)—as are various philosophers who are associated with the logical empiricist movement but who stayed in Europe or were born in the United States (e.g. Quine, nr. 5; Ayer, nr. 10 Nagel, nr. 27; and Schlick, nr. 46). In the top 50, we only find one migrant from the Neo-Kantian tradition (Cassirer, nr. 30), and no migrants representing phenomenology or critical theory. Two main figures of the phenomenologist tradition (Husserl and Heidegger) are included lower on the list (nrs. 19 and 23), but they did not emigrate.

### Mention statistics tracking temporal shifts in intellectual success

**Fig. 7:**
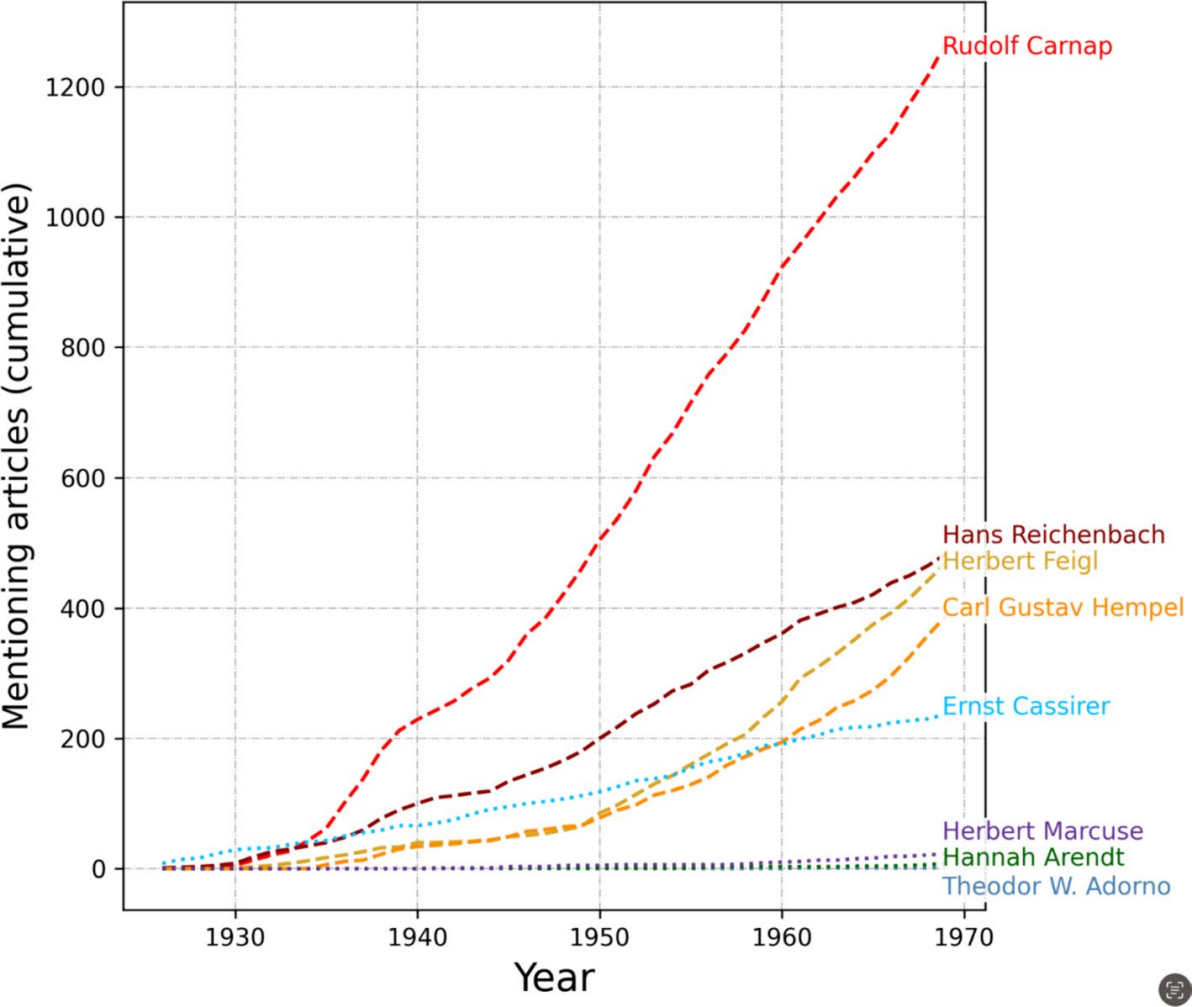
Cumulative number of mentions for eight philosophers 1926–1970. Logical empiricists are represented by dashed lines in warm colors, other important philosophers by dotted lines in cooler colors

EDHIPHY allows also more fine-grained quantitative assessments. We can look at the success of logical empiricist philosophers over time (this subsection), or per institution (subSect. “Comparative analysis to differentiate reception at different institutions”). First, let us take a closer look at the most-mentioned logical empiricists from Table 7 (Carnap, Reichenbach, Feigl, and Hempel) and analyze the numerical growth of their distinct mentions between 1926 (before the migration) and 1970 (when analytic philosophy had achieved a dominant status in the USA). As a contrast case, we will also do the same for four other important philosophical migrants from different traditions, viz. Theodor W. Adorno, Hannah Arendt, Ernst Cassirer, and Herbert Marcuse. Figure 7 shows the cumulative number of articles that mention these philosophers.

This quantitative comparison reveals that Carnap is by far the most successful migrating philosopher in the professional US philosophy world, already from the late 1930s onward. Combined, the four logical empiricist migrants receive 9.66 times more mentions than migrating philosophers from competing traditions (Marcuse, Adorno, Arendt, Cassirer). Interestingly, mentions to Cassirer stay on par with the logical empiricist migrant Hans Reichenbach in the 1930s and 1940s and Cassirer even remained a source of interest in the 1950s and 1960s. In stark contrast, Arendt, Marcuse and Adorno received few mentions in US philosophy journals throughout these decades in comparison, in spite of their impact as public intellectuals, such as Arendt’s widely reverberating coverage of the Eichmann trial or Marcuse’s widespread popularity during the student protest movement of 1968. Of course, these migrated philosophers could have more mentions in other professional, academic circles (such as psychology, sociology, or literature studies). However, this again illustrates how the reception of migrants in professional philosophy was heavily skewed toward the logical empiricist movement.

### Comparative analysis to differentiate reception at different institutions

EDHIPHY also allows aggregate mention-statistics per institution. Currently, mentioning authors and mentioned authors can be associated with an academic institution if they submitted their PhD at a US university or were employed at Harvard, Stanford, Berkeley, UCLA, University of Michigan, University of Pennsylvania, Cornell, Columbia, University of Chicago or Yale at one point between 1940 and 1970.

Some historians have suggested that Harvard played a special role in the reception of logical empiricism, as this is the university where Lewis and Quine were based and where Feigl started promoting the movement after leaving Europe in 1930 (Isaac, 2005; Verhaegh, 2020c). Other universities, like Chicago, seem to have been much more hostile to logical empiricism as they sought to preserve a religious/idealist approach to philosophy (Reisch, 2005). EDHIPHY can shed light on potential differences between these American philosophy departments in their reception of migrating philosophers. In Table 8, we show for the 1950s which philosophers are most mentioned by those authors who were educated or employed by one of six prestigious departments.

**Table 8:** Top 15 most-mentioned philosophers in six American journals 1951–1960 by institutional background of authors. For each philosopher, the number of mentioning articles is reported. An article published in year $\text{y}$ was assigned to a department $\text{d}$ iff (a) one of the authors was employed by $\text{d}$ in $\text{y}$ or $\text{y}-1$ or (b) one of the authors obtained their Ph.D. at $\text{d}$ between $\text{y}-3$ and $\text{y}+3$. Philosophers marked with an asterisk (*) are considered historical authors, because they were born before 1850, those with the question mark (?) are false positives

Rank	Harvard		Yale		Princeton	
1	Bertrand Russell	32	Immanuel Kant*	18	Rudolf Carnap	18
2	Aristotle*	28	Rudolf Carnap	16	David Hume*	10
3	Plato*	26	Plato*	14	Nelson Goodman	9
4	Rudolf Carnap	23	David Hume*	13	Willard V. O. Quine	9
5	Socrates*	22	Alfred N. Whitehead	12	Bertrand Russell	8
6	David Hume*	21	Bertrand Russell	12	Plato*	8
7	Willard V. O. Quine	21	Aristotle*	11	Herbert Feigl	8
8	Immanuel Kant*	20	Georg W. F. Hegel*	10	Socrates*	7
9	John Dewey	16	Willard V. O. Quine	9	Aristotle*	7
10	John Stuart Mill*	16	Socrates*	9	H. H. Price	7
11	Nelson Goodman	16	Gottfried W. Leibniz*	8	Carl G. Hempel	7
12	Clarence I. Lewis	16	George Berkeley*	8	Wilfrid Sellars	7
13	G. E. Moore	15	René Descartes*	7	Ludwig Wittgenstein	7
14	Alfred N. Whitehead	14	Lewis W. Beck	7	Alonzo Church	6
15	René Descartes*	14	Wilfrid Sellars	7	C. D. Broad	6

Rank	Columbia		Chicago		Berkeley	
1	John Dewey	28	Aristotle*	20	Rudolf Carnap	9
2	Plato*	24	Immanuel Kant*	19	Bertrand Russell	6
3	Aristotle*	21	Plato*	15	Gottlob Frege	6
4	Immanuel Kant*	18	David Hume*	15	George Berkeley*	5
5	David Hume*	14	Alfred N. Whitehead	13	Ludwig Wittgenstein	4
6	John Stuart Mill*	13	John Dewey	11	P. F. Strawson	4
7	Socrates*	12	Rudolf Carnap	10	Willard V. O. Quine	4
8	Georg W. F. Hegel*	12	Willard V. O. Quine	9	Albert Einstein	4
9	René Descartes*	12	Gottfried W. Leibniz*	8	Alfred Tarski	3
10	Bertrand Russell	10	Charles S. Peirce*	8	G. E. Moore	3
11	Benedictus de Spinoza*	9	René Descartes*	8	George Santayana	3
12	Rudolf Carnap	9	John Locke*	7	David Hume*	3
13	Alfred N. Whitehead	9	F. H. Bradley	7	Alonzo Church	3
14	Charles S. Peirce*	8	Socrates*	7	Robert du Var?	2
15	William James*	8	Wilfrid Sellars	7	Ernest Nagel	2

Columbia, Yale, Harvard and Chicago are notably different than Princeton and Berkeley. First, in the top 15, there are more historical authors mentioned by scholars trained or employed at Columbia and Chicago in comparison to the other institutions. (Columbia: 9 historical authors in the top 15, Yale: 9, Chicago: 8, Harvard: 8, Princeton: 4, Berkeley: 2). This is in line with the expectations in the literature that the history of philosophy was more important at these institutions than discussions of contemporary philosophers. Second, at Columbia, Yale, Harvard and Chicago, of all migrating logical empiricists only Rudolf Carnap appears in the top-15 mentioned authors and at a lower rank compared to Princeton and Berkeley, which illustrates that logical empiricist philosophy did not receive the same scholarly attention there as in the other institutions. Surprisingly, this stands in opposition to the image in the secondary literature of Harvard as the bridgehead for logical empiricist philosophy. Also notable is Carnap’s position as the most-mentioned philosopher in papers from Princeton and Berkeley philosophers in the 1950s, eclipsing even major historical philosophers such as Kant, Aristotle or Hume. This again confirms the great influence of his work for the American philosophy profession at the time.

### Co-mention analysis

As we noted in the introduction, the linkage data offered by citation indices stand at the core of powerful methods, such as bibliographic coupling and co-citation analysis, that allow detailed and extensive mapping of the structure and dynamics of scientific disciplines (Börner, 2010; Chen, 2013; Petrovich, 2021b). The mention index allows the extension of these methods to past periods of science and scholarship, with the only difference that mentions instead of citations are used to build the network. In this final section, we will exploit in particular *co-mention networks*, which are the natural counterparts of classic co-citation networks (Small, 1973). In a co-mention network, nodes represent the chosen unit of analysis (in our case, philosophers covered in EDHIPHY) and links the number of documents in which the two units are mentioned together. Specifically, co-mention relations can be summarized a matrix with a row and a column for each unit of analysis. The element ${c}_{i,j}$ then represents the number of articles in which both units *i* and *j* are mentioned.

In a co-mention network, we expect philosophers that are frequently mentioned together to cluster and form communities within the network. If these communities can be interpreted in terms of shared intellectual traits (common research area, common approach, common topic, etc.), then co-mention networks can be a valuable tool for mapping the intellectual landscape of a field and track its evolution over time.

To test this hypothesis, we generated two co-mention networks from two corpora of articles covered by EDHIPHY. The first network is extracted from the 1274 articles published in our selection of American philosophy journals in the period 1910–1919. The second network from the 1881 articles published in the period 1950–1959. The first network, therefore, should reflect the intellectual landscape of philosophy in America before the intellectual migration and the rise of analytic philosophy; whereas the second network should reflect how the situation changed after these two major events.

**Fig. 8:**
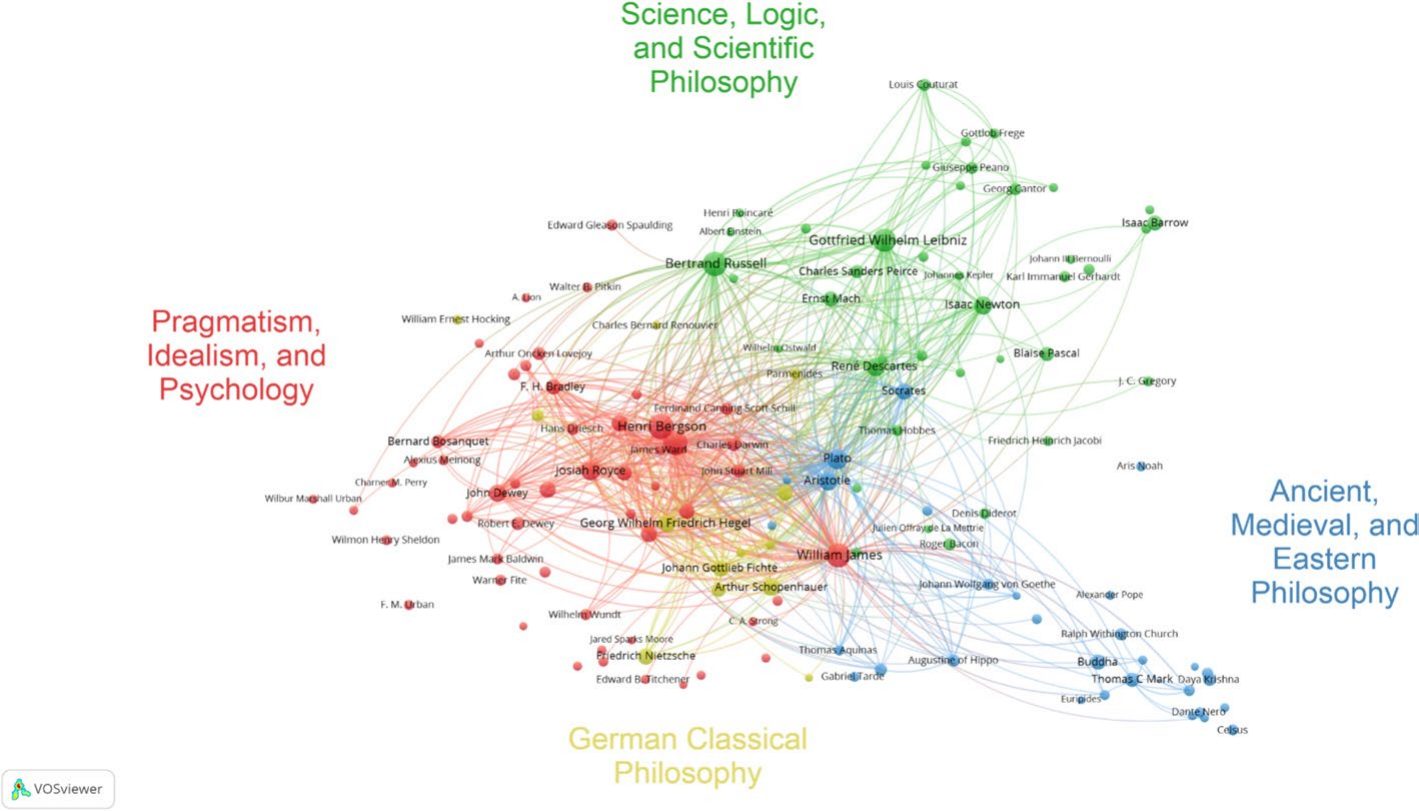
Co-mention network of philosophers mentioned in articles appeared in American philosophy journals in the period 1910–1919. Threshold for inclusion on the map: 50 mentions; Only links with strength 10 or more are shown. Resolution parameter of the clustering algorithm = 0.9. The interactive visualization is available at https://tinyurl.com/25du9nl3

**Fig. 9:**
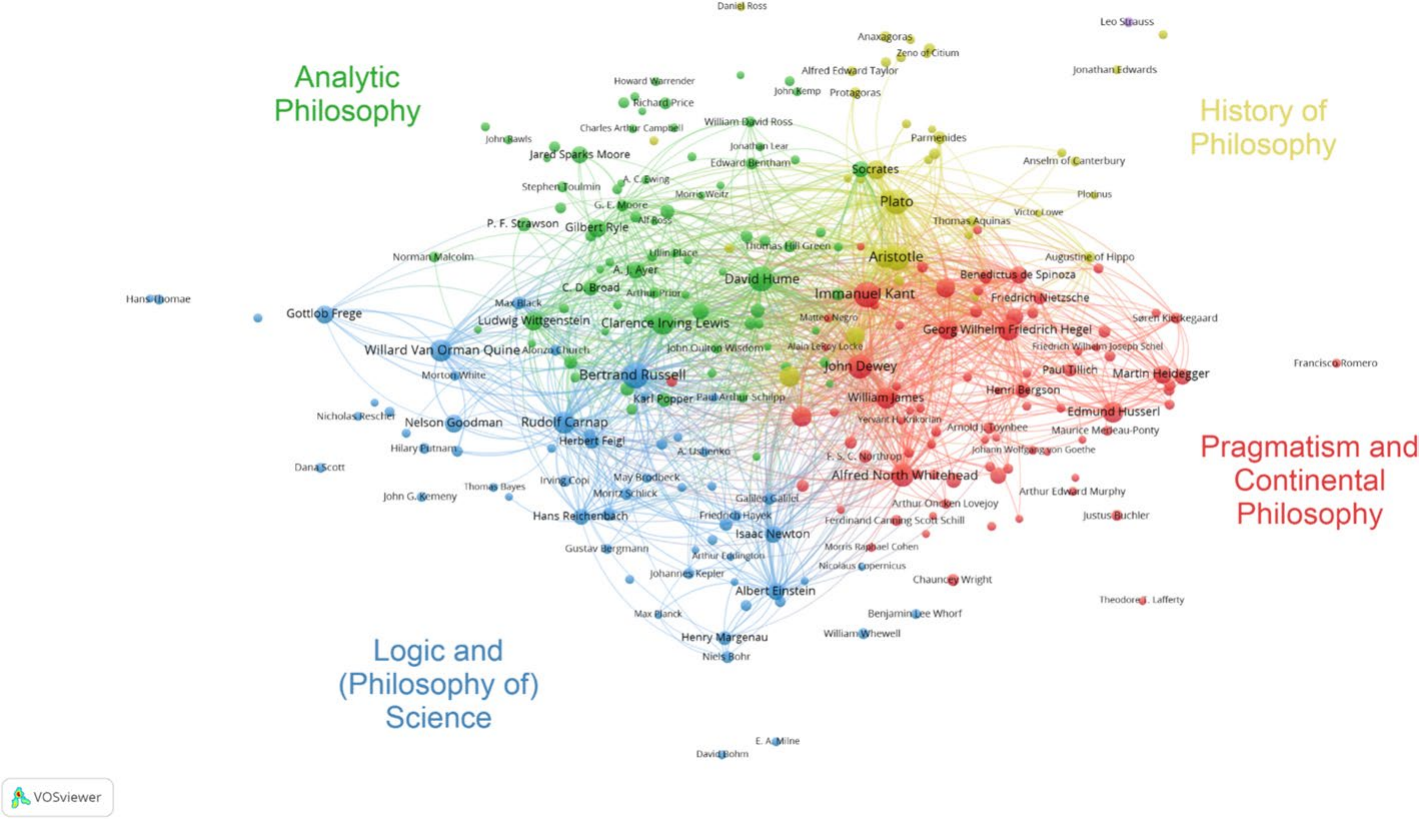
Co-mention network of philosophers mentioned in articles appeared in American philosophy journals in the period 1950–1959. Threshold for inclusion on the map: 50 mentions; Only links with strength 10 or more are shown. Resolution parameter of the clustering algorithm = 0.9. The interactive visualization is available at https://tinyurl.com/2xhkndhe

Figure 8 and Fig. 9 show the two co-mention networks, focusing on philosophers that received at least 50 mentions in the respective corpus of articles (n = 137 and n = 257, respectively). Visualizations are produced with VOSviewer (van Eck & Waltman, 2010) and, as usual, the position of the nodes on the map reflect the co-mention similarity between nodes, so that frequently co-mentioned philosophers will appear close on the map whereas seldom co-mentioned philosophers far apart. The color represents the cluster each node is attributed to based on VOSviewer’s clustering algorithm (Waltman et al., 2010). Lastly, the size of the nodes is proportional to the number of mentions a philosopher received in the corpus of articles considered.

Interestingly, in both maps the groups of philosophers identified by the clustering algorithm do share some common trait. In the 1910s map, the green northern cluster includes philosophers with interests in formal methods, logic, and science (Leibniz, Bertrand Russell, Louis Couturat, Gottlob Frege), the blue cluster various philosophers from ancient and medieval periods (Plato, Aristotle, Augustine of Hippo), as well as religious figures (Buddha), the yellow cluster exponents of German classical philosophy (Hegel, Fichte, Schopenhauer, Nietzsche), and, lastly, the red cluster various representatives of pragmatism (William James, John Dewey) and several psychologists and philosophers interested in psychological phenomena (Edwin Holt, Wilhem Wundt, Edward Titchener), attesting the close connection between psychologists and pragmatists at the beginning of the century (Pearce, 2020).

In the 1950s map, the clusters match even more closely standard groupings of philosophers individuated by historians of philosophy via standard, qualitative methods. Starting from the northern side of the map and moving in counterclockwise direction, the green cluster comprises mainly philosophers from the British tradition (David Hume, John Stuart Mill), including representatives of the British branch of analytic philosophy, focusing on the analysis of ordinary language (Gilbert Ryle, P. F. Strawson); the blue cluster includes philosophers belonging to logical empiricism (e.g., Rudolf Carnap, Hans Reichenbach, Carl Gustav Hempel) and the other variant of analytic philosophy, focusing on formalized language (e.g., Bertrand Russel) and logic (e.g., Alfred Tarski), as well as physicists (Albert Einstein and Niels Bohr) and scientists-philosophers (Henri Poincaré), who contributed to the revolutionary physics theories of the beginning of the century, relativity theory and quantum mechanics, which were widely discussed in American philosophy (Verhaegh, 2024); the red cluster includes several representatives of pragmatism and traditional American philosophy (e.g., William James, John Dewey, and Charles Sanders Peirce) as well as figures that are nowadays frequently gathered under the umbrella term of “Continental” philosophy, including representatives of phenomenology (Martin Heidegger), existentialism (Soren Kierkegaard, Jean-Paul Sartre), German Idealism (Hegel) and other European philosophical traditions; lastly, the yellow cluster comprises several major philosophers from ancient, medieval, and early-modern philosophy—it is therefore likely to interpret this cluster as representing the history of philosophy, as it is also shown by the presence of several philosophers from Ancient Greece in the outer skirt of the cluster.

The two maps reflect the major change of the philosophical landscape between the two periods: in particular, analytic philosophy and logical empiricism were absent in the first map but occupy at least one third of the second map. Coupled with the mention analyses of the previous section, the co-mention networks allow to track with quantitative methods the impact of these changes in twentieth century philosophy. More generally, the fact that the clusters in co-mention networks capture groups of philosophers that share common intellectual traits, such as belonging to the same tradition or research area, demonstrates that co-mentions, similarly to co-citations, capture quite effectively underlying intellectual structures.

## Conclusion

The above examples only provide rough quantitative analyses of the changes that occurred in philosophy during the first half of the twentieth century, but they showcase EDHIPHY’s potential as a database, and the potential of mention analysis overall. EDHIPHY offers philosophers and historians the unique ability to track mentions to historical and contemporary scholars in historical academic writing which does not have standardized citations. A particular strength of the database is that it connects these mention-statistics with further metadata. Examples 2 and 3 show how mention statistics can be aggregated over time or institution. EDHIPHY also allows to visualize the relations between different philosophers by creating co-mention networks. These are only some indicative examples; we believe that many more historical questions can be operationalized in a way that EDHIPHY can help to answer them (see e.g. Verhaegh et al. manuscript for a more detailed analysis). To this end, we will launch https://edhiphy.org on 21st August 2024. This web-application allows anyone to create their own analyses, without requiring technical training, while technical users can also interact via SQL queries. We will also collect feedback and corrections for the further development of EDHIPHY.

### Concluding remarks

Standard citation-based bibliometric tools have severe limitations when they are applied to (1) periods in the history of science and scholarship before the advent of now-current citation practices, (2) academic disciplines with atypical publication or citation cultures. This paper has presented an alternative method—*the extraction and analysis of mentions*—to map and analyze links between scholars and publications in periods and fields that fall outside the scope of citation-based studies. Focusing on one specific discipline in a single period and language area—Anglophone philosophy between 1890 and 1979—we described a procedure to identify, extract, and disambiguate mentions in academic publications. This procedure allowed us to create a *mention index* that includes 1,095,765 mention links, extracted from 22,977 articles published in 12 academic journals. Our disambiguation methodology, based on four distinct strategies, successfully linked 93% of these mentions to specific philosophers, with an estimated precision of 82% to 91%. In addition, we integrated the mention index into a database, called EDHIPHY, which includes, in addition to mention links, several other data and metadata from multiple sources, allowing rich, multidimensional mention analyses. In the final part of the paper, we presented an extended case study demonstrating the use and the potential of both EDHIPHY and mention analyses more generally. In this section, domain experts illustrated how the database can be used to answer open questions about the structure and development of an academic discipline in an innovative way.

Compared with standard, citation-based analyses, mention analysis has the important advantage that it is applicable to most historical periods and scholarly areas where references are not formatted as citations. For most of the history of science, scholars have referenced their peers and predecessors by mentioning them by name (Small, 2010). The modern bibliographic citation is a nineteenth century invention (Bazerman, 1988; Connors, 1999) and in humanistic fields it is still relatively common to mention peers instead of citing specific documents. This is especially true when dealing with canonical or paradigmatic figures and/or when authors assume familiarity with specific philosophical ideas among their readers.

Still, mention analysis is not just an important tool for analyzing academic literature with non-standard citation norms. It can also be used to analyze more recent academic literature as it has several potential advantages over citation-based studies. For one thing, mention-based analyses typically employ a larger amount of information about links between publications than citation-based analyses. Since every citation includes at least one mention but not all mentions are citations, citation-based studies throw out valuable data that can help scholars map and analyze links between publications. A second advantage is that mention-based research is likely to generate more valid results when combined with keyword or sentiment analyses which rely on the textual context in which words are used. Though citation contexts are frequently analyzed in bibliometric research, citations are always separated from the main text to some degree. They are included in footnotes and in bibliographies or, in the case of in-text citations, are typographically and grammatically separated from the scholarly text. Mentions, on the other hand, are usually an element of the grammatical structure of the scientific text itself. This allows one to easily integrate mention analyses with more concept-focused analyses such as term co-occurrence or concordance analyses.

In addition to these advantages, mention-based analyses also face a number of technical challenges. Some of these have been identified in this paper. First, they demand high-quality textual data, unlike text analysis methods relying on a bag-of-word approach such as classic topic-modelling (Sect. “Preparation of texts”). Second, standard named entity recognition systems appear to perform rather poorly in extracting mentions, at least when applied to the corpus studied in the present paper, such that the quality of one’s mention-extraction depends on the quality and completeness of one’s entity ruler (Sects. “Creation of the entity ruler”-“Linking mentions to philosophers”). Third, since mentions are not standardized and contain less information than most citations (often just a surname), one needs an elaborate strategy to uniquely link mentions to authors. The present paper has presented several strategies to extract and disambiguate mentions and to improve mention linking, evaluating each of these strategies by manually checking the accuracy of our mention links (Sect. “Disambiguation of mentions”).

Citation-based analyses, we have seen, ignore valuable information because they disregard references that are not formatted as standard citations to other documents. The mention-based analyses presented in this paper, however, also throw out information because they are exclusively focused on authors. In developing a mention index of twentieth-century Anglophone philosophy, we have focused on the extraction and disambiguation of mentions to individual philosophers, thereby ignoring alternative sources of information, such as mentions of titles, journals, publication years, or even philosophical schools. Note though that this is not an inherent feature of mention analysis. Theoretically, one could use a similar procedure to identify mentions of entities other than authors. A Plato scholar, for example, might expand EDHIPHY by identifying mentions to specific dialogues such as the *Theaetetus* and the *Gorgias*. Indeed, EDHIPHY has been set up in such a way that it allows for easy extension of the mention index. We already included data about gender, birth years, graduate education, and career paths (Sect. “Enriching the mention index”). In the future, we aim to add topic models, keyword analyses, and to expand EDHIPHY in space and time, including data on philosophy outside the Anglophone world in a broader range of historical periods. We will also rely on the feedback for edhiphy.org to guide the further development of the database and its web-application.

Furthermore, the data contained in EDHIPHY and its successors will be pivotal for developing a full-fledged *theory of mentions* in scholarly fields. Such a theory is needed to shed light on the differences between mention and citation links, on their contribution to the circulation of prestige in scholarly fields, and on their role in the construction of scholarly knowledge and communities. Moreover, a full-fledged history and theory of mentions is required to fully assess the advantages and limitations of mention analyses. EDHIPHY, however, can be seen as a first step toward developing such a theory, much as Garfield’s citation index helped fuel theoretical work on the function of citations in the 1960s.

## Footnotes

1 In the last decade of the nineteenth century, it was still common to find the following type of reference in prestigious journals such as *Nature*: “It appears to me that Prof. Weldon’s argument, referred to in *Nature* of July *16* (p. 245), is accurately represented in the following illustration” (Cunningham 1896, cited in Pence, 2022) – a form that is entirely different from current citations.

2 Note that many citations contain mentions, in so far as the cited references include the names of the authors of the cited documents. However, there can also be citations that do not include mentions, such as the citations that refer to documents without a specific author, like the Bible.

3 Journals were selected based on our domain expertise, as several authors of the present paper work on the history of twentieth-century philosophy. We highlight that the entire process of creation of EDHIPHY’s mention index was supervised by historians of philosophy to ensure the historical reliability of the database.

4 After 1980, Web of Science starts covering citations in some of these journals (Petrovich & Buonomo, 2018).

5 Structured representations of texts, such as the.xml files generated in accordance with the Text Encoding Initiative standards (https://tei-c.org/) would be the ideal option for achieving fully reliable and more sophisticated mention extraction. Unfortunately, they are not available for our corpus.

6 Comparable results were obtained using the NER algorithms of other packages.

7 Several tools for training NER algorithm are available in the spaCy environment. See for instance *prodigy* (https://prodi.gy/) [accessed 28.09.2023].

8 SpaCy model of English language, for instance was trained on over 2 million words, so in order to completely change the definition of an entity type, a considerable number of training examples is required (see https://spacy.io/usage/rule-based-matching#entityruler). As we will see below, manual training potentially solve the second task in the building of the mention index, the individuation of mention, but does not address the third task, that of linking mentions to specific philosophers.

9 The impact of such false positives is discussed in Sect. "Evaluation of linking" below.

10 https://www.wikidata.org/wiki/Wikidata:Main_Page

11 The query is available at https://w.wiki/6UBe. Data were retrieved on 21.03.2023.

12 https://en.wikipedia.org/wiki/Donald_Davidson_(philosopher).

13 JSTOR was the most problematic dataset in this sense. A few duplications were individuated in WikiData as well, however.

14 A case of spurious match is the match between the WikiData entry Bertrand Russell and the JSTOR author “B. Roswell Russell”, which derives from the match with the variant “B. Russell”.

15 A special script was developed for parsing author strings and derive an “artificial surname”. The script was able to manage also the most common surname particles such as “Von” or “De”.

16 This time, since the JSTOR authors list was significantly cleaner, fuzzy matching between strings was applied instead of clustering.

17 A small number of variants ($n=44$) were manually added after inspection of the results to improve the recall of mentions of some specific philosophers.

18 The longer the list of philosophers, the longer the list of variants and the higher the chance of one-to-many relationships between mentions and philosophers.

19 Articles without an author (3% of the corpus) were excluded from this strategy. Articles with more than one author, which thus belong to multiple *oeuvres*, were used as context of reference only once (i.e., they were included in the *oeuvre* of only one author), in order to avoid potential conflicts in the disambiguation process.

20 Note that the number of mentions include both the univocal mentions and the mentions successfully disambiguated through strategies one and two.

21 Note that, differently from a classic co-citation matrix, the co-mention matrix is *not* symmetric, i.e., ${c}_{i,j}$ can be different from ${c}_{j,i}$. This happens because the same philosopher can be mentioned multiple times in the same article. For instance, let $A=[Plato, Aristole, Plato]$ be the list of mentions occurring in paper $A$. In this scenario, Plato is co-mentioned twice with Aristotle because Plato is mentioned two times in paper $A$, where Aristotle is also mentioned. By contrast, Aristotle results to be co-mentioned only once with Plato, because, in the same paper $A$, Aristotle is mentioned only once. If the metric used were the number of co-mentioning *articles*, the co-mention matrix would be symmetric. In terms of performance of the disambiguation strategy, however, there is no difference between the two.

22 Clearly, the mention vectors of the articles were extended to match the length of co-mention vectors of the philosophers in order to make the computation of the cosine possible. The extra components, properly matched, were set to 0. For instance, if the co-mention vector $\overrightarrow{P}$ included a non-zero value for the component ${c}_{w}$ encoding the co-mentions of philosopher $w$ with $P$ but $w$ is never mentioned in paper $r$, then the mention vector $\overrightarrow{r}$ was extended with a component ${c{\prime}}_{w}$ that was set to 0 in order to make the computation of the cosine between $\overrightarrow{P}$ and $\overrightarrow{r}$ possible. We also tested an alternative approach where only the components occurring *both* in the mention vector and in the co-mention vector were considered in the computation of the cosine similarity, but the precision of the disambiguation resulted lower.

23 The article "Sellars' Theory of Valuation" (http://www.jstor.org/stable/2103741) is a real example of such an article, where the first name of the mentioned Sellars is never used by the author.

24 The birth year was obtained from WikiData and, clearly, was available only for philosophers that are present in WikiData.

25 These lists will undergo continuous updates in the future to further enhance the database’s quality.

26 Olensky and colleagues (2016) note that “little is known” about the citation matching algorithm employed by Web of Science, even if it seems to be rather conservative and not allow for any variation in the cited reference strings.

27 Mention numbers reflect the number of distinct articles in which a philosopher is mentioned. We included publications in six American journals of philosophy (*Philosophical Studies*; *Philosophy and Phenomenological Research*; *Philosophy of Science*; *The Journal of Philosophy*; *The Monist*; and *The Philosophical Review*) from 1951 to 1960.
